# Effect of different killing methods during curing on the phytochemical and bacterial composition of *Vanilla planifolia* using multi-omic approaches

**DOI:** 10.1016/j.fochx.2025.102269

**Published:** 2025-02-08

**Authors:** Tiffany A. Cuan-Escobar, Alma Cuellar-Sánchez, Haiku D.J. Gómez-Velázquez, Juan L. Monribot-Villanueva, José A. Guerrero-Analco, Isabel Gutiérrez-Díaz, Diego A. Luna-Vital

**Affiliations:** a Tecnologico de Monterrey, The Institute for Obesity Research, Ave. Eugenio Garza Sada 2501, 64849 Monterrey, NL, Mexico; b Tecnologico de Monterrey, School of Engineering and Science, Ave. Eugenio Garza Sada 2501, 64849 Monterrey, NL, Mexico; c Departamento de la Tierra y de la Vida, Centro Universitario de los Lagos, Universidad de Guadalajara, C.P. 47460 Lagos de Moreno, Jalisco, Mexico; d Laboratorio de Química de Productos Naturales, Red de Estudios Moleculares Avanzados, Instituto de Ecología, A. C., Carretera Antigua a Coatepec 351, 91073 Xalapa, Veracruz, Mexico; e Department of Microbiology and Biochemistry of Dairy Products, Instituto de Productos Lácteos de Asturias (IPLA), Consejo Superior de Investigaciones Científicas (CSIC), Villaviciosa, Asturias, Spain

**Keywords:** *Vanilla planifolia*, Curing process, Killing process, Phenolic compounds, 16S sequencing, Metabolomics

## Abstract

*Vanilla planifolia* Jacks. ex Andrews, is cultivated for its aromatic pods, obtaining the primary source of vanillin, a molecule valued for its flavor and bioactivity. Mexico ranks among the top five global producers, and Papantla, Veracruz, contributes 70 % of national production. Developing vanilla's characteristic aroma involves a curing process composed of killing, sweating, drying, and conditioning, which enzymatic reactions and microbial activity play essential roles. This study assessed the impact of four killing treatments: microwave, hot water immersion, sonication, and freezing on the phenolic composition and bacterial communities in vanilla curing through metabolomic and 16S sequencing approaches. Freezing treatment resulted in the most substantial changes in phenolic profiles, including higher vanillin concentrations. *Bacillus* was the dominant bacterial genus, with hot water immersion and sonication showing the greatest α-diversity. These findings underscore the value of omic sciences in refining curing processes, enabling producers to achieve higher-quality vanilla through more efficient and technical methods.

## Highlights

• Vanilla killing methods impact phenolic compounds and bacterial populations.
• Freezing treatment yields the highest concentration of vanillin.
• *Bacillus* genus is the main bacterial population in cured vanilla pods.
• *Bacillus* had higher positive correlation with glucovanillin, a key precursor of vanillin.
• Killing before curing modifies bacterial population, curing gets high diversity.

## Graphical abstract

**Figure:**
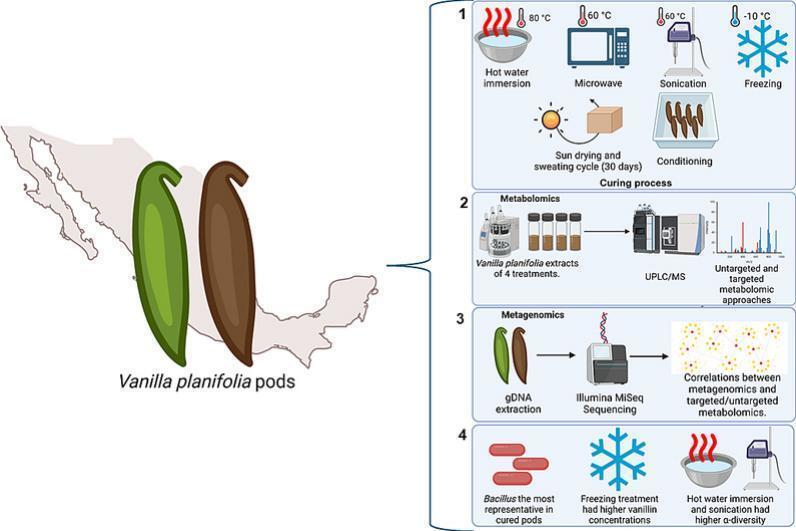

## 1 Introduction

*Vanilla planifolia* Jacks. ex Andrews (Ramos-Castellá et al., 2016), a member of the *Orchidaceae* family, is the only orchid cultivated for human consumption due to its production of an aromatic fruit: the vanilla pod (Ahmad et al, 2020; Baqueiro-Peña & Guerrero-Beltrán, 2017). According to the office of agriculture in Mexico (Secretaría de Agricultura y Desarrollo Rural) (SADER., 2023) and Barrientos et al. (2023), Mexico is positioned among the top five main vanilla producing countries, leading Madagascar contributing 43.9 % of the total output. In Mexico, the city of Papantla, Veracruz, is recognized as the center of vanilla cultivation, production, and sale, accounting for 70 % of the national production, followed by Puebla and Oaxaca (30 %) (Baqueiro-Peña & Guerrero-Beltrán., 2017).

The primary molecule in *V. planifolia* is vanillin, a key component of commercial vanilla extract, which ranges from 0.75 to 4.58 % concentration in cured pods (Barrientos et al, 2023; Pérez-Silva et al, 2021; Arya et al, 2021). This molecule is highly valued not only for its role in food flavoring but also for its bioactive properties, including anticancer, neuroprotective, antibiotic-potentiating, and anti-quorum sensing activities (Arya et al., 2021). Green vanilla pods lack the characteristic aroma of cured pods because phenolic compounds associated with the aroma are present as glucoside conjugates (Dignum et al., 2002). These sensory attributes develop during the curing process, which involves several enzymatic and chemical reactions, including the release of aglycones by β-ᴅ-glucosidase (Pardio et al., 2009).

The curing process includes four stages: killing, sweating, drying, and conditioning. Killing, the first stage, is crucial as it induces senescence and subcellular decompartmentalization, enabling enzyme-substrate reactions that produce aroma compounds (Mariezcurrena et al., 2008). Methods such as hot water scalding, sun and oven wilting, and freezing are commonly used for killing (Anuradha et al., 2013). These methods were selected based on their frequent use in experimental studies reported in the literature, where they have shown promising results in enhancing the production of vanillin and other phytochemical compounds that serve as vanillin precursors (Pardio et al, 2009; Mariezcurrena et al, 2008; Antonio-Gutiérrez et al, 2023). During the sweating stage, pods are kept in high-humidity conditions within mahogany boxes to catalyze oxidative and hydrolytic reactions that develop the vanilla's aroma, and color while preventing microbial deterioration. This stage is cyclically combined with sun drying for 5 to 30 d, which facilitates enzymatic activity and gradual dehydration necessary for aroma development (Zamora-Flores, 2015; Baqueiro-Peña & Guerrero-Beltrán, 2017). Drying, however, can be uneven due to variability in pod size and environmental conditions (Zamora-Flores., 2015). Finally, in the conditioning stage, dried pods are stored at room temperature for 3–4 months to undergo chemical and biochemical reactions like esterification, etherification, and oxidative degradation, which enhance aroma quality (Anuradha et al., 2013). Even though in traditional approaches the conditioning takes several months, in this study we used one week, mainly due to previous studies in literature. According to Dignum et al. (2002), they carried out the curing of green vanilla pods at scale and under laboratory conditions, considering short conditioning times for this project. In addition, it has been found that at one week of conditioning, they obtained a higher concentration of glucovanillin (2060 ppm), which is the main precursor of vanillin (Dignum et al., 2002). The purpose of the study of different killing treatments is that it can help in process efficiency, time and cost reduction, and in the sustainability of the processes with less environmental impact, guaranteeing quality products satisfying the demand for natural vanilla in the national and international market.

The phytochemical composition of vanilla pods depends on factors such as species, environmental conditions, and curing methods (Baqueiro-Peña and Guerrero-Beltrán., 2017). For instance, Barreda-Castillo et al. (2023) used targeted metabolomics to identify phenolic compounds in *V. planifolia* seedling leaves, including vanillin, vanillic acid, and *trans*-cinnamic acid, compounds commonly found in cured pods.

During the curing process of vanilla, high temperatures and gradual dehydration induce significant changes in the composition and structure of the pods, creating an environment where microbial communities can utilize available carbon sources, thereby contributing to flavor development (Barrientos et al., 2023). Microorganisms such as *Terribacillus*, *Pseudomonas*, *Luteibacter*, and *Bacillus* have been identified at various stages of processing and exhibit enzymatic activities, including amylolytic, pectinolytic, and cellulolytic functions, which are closely linked to the physicochemical transformations occurring during curing (Barrientos et al., 2023). These microbial activities, combined with the enzymatic processes of the vanilla pods themselves, play a critical role in the synthesis of aromatic compounds, highlighting the intricate relationship between microbial dynamics and the physicochemical parameters experienced during processing (Barrientos et al., 2023).

The objective of employing a combined targeted and non-targeted metabolomic approach was to quantify and identify both key metabolites and secondary compounds present in vanilla extracts. This allowed us to evaluate differences in phytochemical profiles between extracts obtained from pods subjected to four different killing treatments. In parallel, the application of 16S sequencing aimed to identify and characterize changes in the bacterial communities present at three specific stages: green pods, pods after killing treatment and cured pods. This integrated approach allows understanding how different techniques affect both chemical composition and microbial populations, providing key information to optimize curing processes and improve the quality of the final product.

Despite its significance, vanilla production in Mexico faces losses of up to 50 % due to pests, diseases, insufficient training of producers, minimal technification, and undervaluation of traditional agriculture (Martínez-Hernández., 2017). Addressing these challenges requires improvements in technification and optimization of curing processes to enhance yield and quality, particularly by focusing on phenolic compounds of interest and leveraging advanced omic technologies. This study hypothesizes that different killing treatments significantly affect the composition of phenolic compounds and bacterial populations in vanilla pods. The objective is to determine the impact of these treatments on the phytochemical and microbial profiles of *V. planifolia,* ultimately contributing to the optimization of curing practices and the development of high-value products.

## 2 Biological material

*Vanilla planifolia* pods (1 kg) were obtained from local producers, in Papantla, Veracruz, whose coordinates are: 20°26′48″N 97°19′30″W / 20.446666666667, −97.325. The pods were collected at approximately 8 to 10 months as suggested by Barrientos et al. (2023). The ripened pods, identified by the yellow coloration of the extreme (Anuradha et al., 2013) were harvested, and approximately 90 to 110 pods were needed to obtain 1 kg of vanilla pods.

## 3 Materials and reagents

The reagents used for targeted and untargeted metabolomics were methanol (Product reference 106,035, Sigma Aldrich), water + formic acid 0.1 % (Product reference 1.59007, Sigma Aldrich), acetonitrile + formic acid 0.1 % (Product reference 576,956, Sigma Aldrich). Used equipment for targeted and untargeted metabolomics were 1290 Infinity Agilent Ultrahigh Resolution Liquid Chromatograph (UPLC, Agilent 1290 series, Santa Clara, CA, USA) coupled to a 6460 Agilent Triple quadrupole mass spectrometer (MS–MS, Agilent 6460, Santa Clara, CA, USA) with a column Zorbax SB-C18 (2.1 × 50 mm, 1.8 μm, Agilent, Santa Clara, CA, USA) and UPLC Class I Waters coupled to Synapt HDMi mass spectrometer (Waters, Milford, MA, USA) and Acquity BEH column (1.7 μm, 2.1 × 50 mm; Waters, Milford, MA, USA), respectively.

The kits used for bacterial DNA extraction, DNA purification and amplification were Quick-DNA Fungal/Bacterial Miniprep (Cat No. D60005, Zymo Research), Wizard SV Gel and PCR Clean Up System (Cat No. A9281, Promega) and DreamTaq PCR Master Mix (2×) (Cat No. K1071, Thermo Scientific) respectively. The sequence primers used for the PCR products were targeted to the V3-V4 region of the 16S gene: Forward 5’-CCTACGGGGGGGGGGGGCWGCAG (Cat No. WD10971557, Sigma Aldrich) and Reverse 5’-GACTACHVGGGGGTATCTAATCC (Cat No. WD10971557, Sigma Aldrich).

## 4 Methodology

### 4.1 Curing process

Before curing, green pods were disinfected with 10 % sodium hypochlorite solution for 10 min and then rinsed 10 times with sterile water. In this study, wounding was used to promote the biosynthesis of polyphenolic compounds in pods. Although wounding is not a common practice in vanilla pods for curing process, according to Yeshi et al. (2022), the abiotic stress factor of wounding stimulates the synthesis of bioactive compounds such as phenolic compounds, compounds of interest in this research. In the literature, it has been found that wounding vanilla pods can be an effective strategy to stimulate the accumulation of bioactive compounds, especially phenolic compounds, considering that these practices are not common in the traditional method (Buitimea-Cantúa et al., 2022). Alternatively, it has been reported that other methods such as the use of high hydrostatic pressures (HHP) can generate stress, breaking the cell wall, favoring enzymatic activity to release precursors of aromatic compounds (Buitimea-Cantúa et al., 2022). In the case of vanilla, Buitimea-Cantúa et al. (2022) reported that HHP significantly increased the content of phenolic compounds in vanilla during the first stages of curing. Four different treatments of killing were used for this study based on Pardio et al. (2009) and Pacheco (2009) which were hot water immersion at 80 °C (80 W) for 10 s 3 times at 30 s intervals, freezing at −10 °C (FREE) for 24 h, sonication at 60 °C (SON) for 3 min at 70 W and microwave (MIC) for 2.5 min at 100 W. In literature, two methods of killing by freezing have been evaluated, either in a − 80 °C freezer or immersion in liquid N_2_. It has been reported that freezing in a − 80 °C freezer is preferable rather than liquid nitrogen due to technical and economic feasibility. However, both treatments are effective and can be utilized depending on the specific requirements of the process (Mariezcurrena et al, 2008; Pardio et al, 2009; Barrientos et al, 2023; Havkin-Frenkel & Belanger, 2018). These methods were selected based on their frequent use in experimental studies reported in the literature, where they have shown promising results in enhancing the production of vanillin and other phytochemical compounds that serve as vanillin precursors (Pardio et al, 2009; Mariezcurrena et al, 2008; Antonio-Gutiérrez et al, 2023).

Each treatment was performed on 28 pods followed by a post-killing treatment by sonication at 38 °C for 5 min at 70 W, this was to ensure an efficient cellular rupture and ensure a higher yield of vanillin (Pacheco., 2009). For drying and sweating process, the pods were exposed to the sun daily (30 d) for 6 h, and subsequently the pods were stored in cotton blankets inside mahogany boxes to sweat at room temperature for the rest of the day. The use of mahogany boxes is a tradition in Papantla, Mexico, mostly by Totonacas indigenous communities, transmitted from generation to generation because it maintains the distinctive organoleptic properties. It also retains heat and humidity, favoring the enzymatic degradation of chemical precursors that generate vanillin (Havkin-Frenkel & Belanger., 2018). Dried pods were conditioned in ventilated mahogany boxes at room temperature for one week, following the protocol of Dignum et al. (2002). Even though in traditional approaches the conditioning takes several months, in this study we used one week, based on previous studies from Dignum et al. (2002), who carried out the curing of green vanilla pods at scale and under laboratory conditions, considering short conditioning times, finding that at one week of conditioning, the pods synthesized a greater concentration of glucovanillin (2060 ppm), which is the main precursor of vanillin (Dignum et al., 2002).

### 4.2 Metabolomic analysis

#### 4.2.1 Phytochemicals extraction

For extraction, the cured pods were ground and passed through a 0.0394 in mesh, and finally subjected to an accelerated solvent extraction system according to the protocol of Luna-Vital & Gonzalez De Mejia. (2018). Briefly, the ASE 350 (Dionex, Sunnyvale, CA, USA) equipment was used for the extraction of compounds of vanilla pods, using methanol 100 % as a solvent. Methanol is a frequently used solvent for metabolomic analyses because it allows to extract polar and non-polar compounds and is preferred because its low boiling temperature facilitating the concentration of the extract. A sample of 3 g of cured grounded vanilla pods and 1 g of diatomaceous sand, were placed into a stainless-steel cell with cellulose filter by triplicate and preheated at 60 °C for 5 min for extraction; the extraction time with static solvent were 15 min. The pressure employed in the cells was 1500 psi with one extraction cycle for the static heating steps. At the end of the extraction, the extracts were purged with nitrogen for 60 s. For the removal of traces of methanol from the extract, the samples were rotary evaporated, freeze-dried, and kept at −20 °C until further use.

#### 4.2.2 Untargeted analysis

A sample of 50 mg of dried extract were weighed for each treatment, and these were dissolved in 1 mL methanol with 1 % formic acid, then the solutions were syringe-filtered at 0.20 μm PTFE membranes.

The chromatographic system was an UPLC Class I of Waters™ coupled to a Synapt HDMi mass spectrometer (Waters, Milford, MA, USA). The chromatography was carried out on an Acquity BEH C18 column (1.7 μm, 2.1 × 50 mm; Waters, Milford, MA, USA) with a column and sample temperatures of 40 °C and 15 °C respectively. The mobile phase consisted of (A) water and (B) acetonitrile, both with 0.1 % of formic acid (CAS No. 75–05-08, Sigma-Aldrich). The gradient conditions of the mobile phases were 0–20 min linear gradient 1–99 % B, 20–24 min isocratic at 99 % B, 24–25 min linear gradient 90–1 % B (total run time 30 min). The flow rate was 0.3 mL/min and 5 μL of extract was injected. The mass spectrometric analysis was performed with an electrospray ionization source in negative and positive (ESI -/+) with a capillary, sampling cone and source offset voltages of 3000, 40 and 80 V, respectively. The source temperature was 120 °C and the desolvation temperature was 20 °C. The desolvation gas flow was 600 L/h and the nebulizer pressure was 650 kPa. Leucine-enkephalin was used as the lock mass (556.2771, [M + H]^+^; 554.2615, [M-H]^−^). The conditions used for MS^e^ analysis were mass range 50–1200 Da, Function 1 CE 6 V, function 2 CER 10–30 V, scan time 0.5 s. The data (spectrometric features composed by the retention time and mass-charge values, R.t-*m/z*) was acquired and processed with MassLynx (version 4.1) and MarkerLynx (version 4.1) softwares (Waters™, Milford, MA, USA). The alignment parameters used in MarkerLynx include an intensity threshold of 500 counts, a mass/charge and retention time tolerances of 0.03 mDa and 0.2 min, respectively. A noise elimination level of 6 was used, isotopic peaks were removed and a 100 % of minimum replicates was set to determine chemical markers. MetaboAnalyst 5.0 software (Xia Lab, McGill University) was used for raw data filtering in both ionization modes and data normalization. For positive ionization mode were used log^10^ transformation and autoscaling. For negative ionization mode were used square root transformation and Pareto scaling. The metabolite annotation (level 3; tentative) was performed with the “Functional Analysis” module of MetaboAnalyst 5.0 using the Mummichog algorithm and the metabolome of *Arabidopsis thaliana* deposited in KEGG as reference. The adducts allowed were [M + H]^+^, [M + Na]^+^, [M-H]^−^ and [M + Cl]^−^ and a mass error below ±5 ppm. The Pathway Analysis was performed with the list of tentatively identified compounds obtained by the Mummichog algorithm and the “Pathway Analysis” module of MetaboAnalyst 5.0 using the *A. thaliana* metabolome deposited in KEGG as reference.

#### 4.2.3 Phenolics-targeted metabolomic analysis

The identification (level 1; validated with standards) and quantification of phenolic compounds was performed as previously reported in Juarez-Trujillo et al. (2018) and Monribot-Villanueva et al. (2019). Samples were dissolved in methanol (50 mg/mL), filtered with 0.5 μm PTFE membranes and placed in 2 mL UPLC vials and the chromatography was performed in an ultra-high performance liquid chromatograph (UPLC, Agilent 1290 series, Santa Clara, CA, USA) coupled to a triple quadrupole mass spectrometer (MS–MS, Agilent 6460, Santa Clara, CA, USA). The mobile phases were water with 0.1 % of formic acid (A) and acetonitrile with 0.1 % formic acid (B), at a flow of 0.3 mL/min and 2 μL of each sample were injected in the system. The column was a Zorbax SB-C18, 2.1 × 50 mm, 1.8 μm (Agilent, Santa Clara, CA, USA) and the column temperature was 40 °C. The mass spectrometer was operated in positive and negative ESI modes with the desolvation temperature of 300 °C, the cone gas (N_2_) flow of 5 L/min, the nebulizer pressure of 310.3 kPa, the sheath gas temperature of 250 °C, the sheath gas flow of 11 L/min, the capillary voltage (positive and negative) of 3500 V and the nozzle voltage (positive and negative) of 500 V. The data was processed with the MassHunter Workstation Software version B.06.00 (Agilent Technologies) and the results are expressed as the average ± standard deviation of μg/g of extract. For identification level 1 (validated), a dynamic multiple reaction monitoring method was developed for 65 different compounds (detailed information in Supplementary Table 1). For quantification, calibration curves were constructed with 13 calibration points from 0.125 to 19 μM obtaining determination coefficients values higher than 0.99 (detailed information in Supplementary Table 1). These data were also analyzed in MetaboAnalyst 5.0, being normalized using square root transformation and autoscaling. The validation of the quantification method of phenolic compounds was carried out examining key parameters including the limits of quantification (LOQ) and accuracy (trueness and precision). The LOQ was determined as the minimum and maximum concentration of the substance of interest that can be reliably quantified obtaining a maximum percent deviation of 20 %. In addition, the coefficient determination of the regression analysis was higher than 0.99 in all cases (Detailed information in Supplementary Table S1). The accuracy of the quantification method was determined selecting 10 phenolic compounds and testing two parameters, the trueness (how far away the result of an experiment is from the true value) and precision (a measure of dispersion). As can be seen in supplementary table 1, the trueness measured as the deviation in percentage between the calculated and expected concentrations exhibited a percent deviation around 20 %. On the other hand, the dispersion measured as standard deviation considering the results of three independent analysis performed in three consecutive days was below 1.31. These results confirm that the accuracy (trueness) and precision (reproducibility) of the analytical method is high.

### 4.3 16S sequencing analysis

#### 4.3.1 Genomic DNA extraction

To analyze potential differences in bacterial populations due to the killing methods, the vanilla samples were divided into three study groups: untreated control (Ctrl), killed green pods (K), and cured pods (C). The control group consisted of two green pods that underwent neither disinfection nor any killing treatment. A total of eight green vanilla pods were subjected to four killing treatments: 80 W, MIC, FREE, and SON. Additionally, eight cured pods from each treatment were included in the experiment (See Supplementary Material 4).

In total, 18 samples with their respective treatments were collected. Each sample was ground into a fine powder using a sterile mortar and pestle under liquid nitrogen (−196 °C) to ensure homogenization while preserving the integrity of the bacterial DNA. Bacterial genomic DNA (gDNA) was then extracted from the ground samples following the protocol provided in the Quick-DNA Fungal/Bacterial Miniprep Kit (Zymo Research).

#### 4.3.2 PCR, purification and quantification of gDNA

Obtaining the gDNA, the amplicon generation was performed using the primers sequences specific to the region V3 and V4 of the 16S gene (F 5’-CCTACGGGGGGGGGGGGCWGCAG and R 5’-GACTACHVGGGGGTATCTAATCC), calculations and protocol for the PCR reaction was followed of the DreamTaq PCR Master Mix (2×) (Thermo Scientific). For PCR products purification, the protocol of Wizard SV Gel and PCR Clean Up System (Promega) was followed. The quantification of final DNA was performed by the NanoDrop One (Thermo Scientific), following the quality specifications: 1.8 to 2.0 for 260/280 ratio and 2.0 to 2.2 for 260/230 ratio.

#### 4.3.3 Illumina MiSeq rRNA 16S sequencing

Primers targeting the variable V3 and V4 regions of the 16S rRNA gene that included the adapter regions compatible with the Nextera XT Index kit (Illumina, San Diego CA, United States) were used to prepare the sequencing libraries. Briefly, for each sample, 5 ng of gDNA mixed with 0.2 μM of Forward Primer (16S Amplicon PCR Forward Primer = 5’TCGTCGGCAGCGTCAGATGTGTATAAGAGACAGCCTACGGGNGGCWGCAG), 0.2 μM of Reverse Primer (16S Amplicon PCR Reverse Primer = 5’GTCTCGTGGGCTCGGAGATGTGTATAAGAGACAGGACTACHVGGGTATCTAATCC), and 1× PrimeSTAR HS DNA Polymerase (Takara, Kusatsu, Shiga, Japan) were amplified using the following PCR method: 1 min at 98 °C, 25 cycles of 30 s at 98 °C, 30 s at 55 °C and 30 s at 72 °C and a final extension of 2 min at 72 °C. PCR products were purified using AMPure XP beads (Beckman Coulter Inc., Brea, CA, United States) in a 0.8:1 bead: sample volume ratio. Index sequences were attached to each library by PCR. For each library, 1 μL of purified amplification product from the V3-V4 PCR was mixed with 1.5 μL of each individual index, 3.5 μL of nuclease free water and 7.5 μL of PrimeSTAR HS DNA Polymerase. Index PCR method was: 1 min at 98 °C, 11 cycles of 20 s at 98 °C, 20 s at 55 °C and 20 s at 72 °C and a final extension of 2 min at 72 °C. Indexed libraries were purified using AMPure XP beads in a 1.12:1 bead: sample volume ratio. Libraries were quantified with Qubit dsDNA HS Assay Kit (Invitrogen, Carlsbad, CA, United States), their size was analyzed in a QSep 400 (BiOptic, New Taipei City, Taiwan), and sequencing was performed in a MiSeq using the MiSeq Reagent kit V3 (Illumina, San Diego CA, United States) in a 301 bp pair-end reads configuration.

Raw data passed by a quality assessment of the metagenomic libraries, performed with FastQC (Babraham Bioinformatics) to determine the quality of the sequencing. All raw sequences passed the initial quality filter. Adapters were removed and a quality and length filter were performed with Trimmomatic 0.40 (Bolger et al., 2014). Kraken2 (Wood et al., 2019) was used to study the taxonomic community compositions of *V. planifolia* microbiota, using the previously processed reads as input. The vegan package was used to calculate the diversity indexes, while ggplot2 was used to produce the stacked bar plots in R (version 3.4.1, R Core Team, 2019) (Oksanen et al., 2020).

### 4.4 Statistical analysis

One way analysis of variance (ANOVA), and a comparison of means with Tukey test post hoc were performed by Minitab 19 statistical package. Data are expressed as the mean ± standard deviation. A value of *p* < 0.05 was considered statistically significant. Correlations between relative abundances of bacterial genera and concentrations of metabolites were performed in Cytoscape software, considering *p* < 0.01 as significant with Pearson correlation values.

## 5 Results and discussion

### 5.1 Untargeted metabolomics

**Fig. 1:**
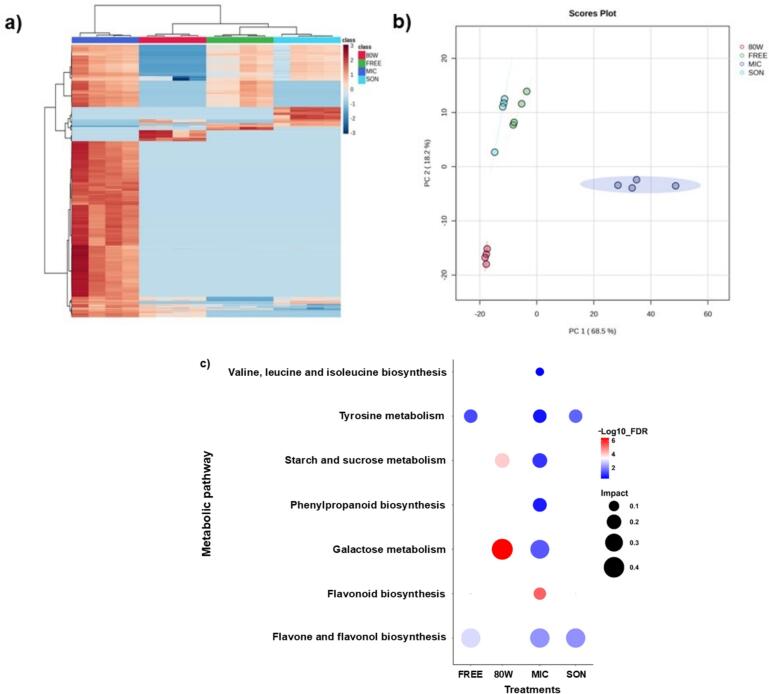
a) Hierarchical clustering heatmap of both ionization modes (ESI + and ESI -) found in untargeted metabolomic analysis as a result of different killing treatments in *V. planifolia* pods. The heatmap was generated using Euclidean and Ward for distance measure and clustering algorithm. Hot water immersion 80 °C (80 W), Freezing (FREE), Microwave (MIC) and Sonication (SON) as treatments. High correlated peak intensities are in MIC treatment. b) Principal Component Analysis (PCA) respecting peak intensities in both ionization modes (ESI + and ESI -) as a result of different killing treatments in *V. planifolia* pods. c) Tentative metabolic pathways found in four killing treatments in *V. planifolia* pods. It was determined by Mummichog algorithm and KEGG database in Functional Analysis module of MetaboAnalyst software. The statistical analysis was made with FDR.

Phenolic compounds are a group of biologically active compounds in plants and are being studied for their potential in health benefits (Abbas et al, 2016; Rasouli et al, 2017). The untargeted metabolomic analysis detected 453 signals (R.t-*m/z*) in positive ionization mode and 2352 signals in negative mode. A large difference between the number of signals detected in positive versus negative ionization mode was found. This could be due to according to Liigand et al. (2017), some compounds have higher affinity for one of the ionization modes depending on their chemical structure, as in the case of basic molecules (amines, amides) tend to ionize better in positive mode, while acidic molecules (carboxylic acids, among other acidic groups) are detected in negative mode. The physicochemical properties of the solvent, such as pH and polarity, have been found to affect the ionization efficiency of compounds in each mode (Kiontke et al., 2016). The names of tentative compounds found in the analysis are listed in Supplementary Table 2. The effect of four killing treatments on the chemical profiles of vanilla pods was evidenced in the following heatmap (Fig. 1a). In microwave (MIC) treatment, was found that most of the R.t-*m/z* signals in both ionization modes (+/−) exhibited higher values of *z-score*, which is a statistical measure that evaluates how far an individual value is from the mean in a data set, in terms of standard deviations (SAS Institute Inc., 2024). The hierarchical clustering allows us to determine that the FREE and SON treatments exhibited similar effect to the vanilla pods chemical composition. The 80 W treatment was similar to FREE and SON treatments, while MIC treatment was the most different (Fig. 1a). The Principal Component Analysis (PCA) (Fig. 1b) explained 86.7 % of the variance and indicated that FREE and SON treatments were clustered in component 2, suggesting similarities in their R.t-*m/z* intensities, which is consistent with the heatmap showed. On the other hand, the 80 W and MIC treatments clustered at different sites on component 1, suggesting differences in their R.t-*m/z* intensities.

The metabolic pathways resulted from the four vanilla killing treatments are presented in Fig. 1c. For more detailed information about the metabolic pathways see the supplementary table 3. Interestingly, both MIC and 80 W treatments impacted the pathway of galactose and sucrose and starch metabolism. Regarding galactose and sucrose pathway, they are not directly involved in the biosynthesis of vanillin, however Yoon et al. (2005) refer to galactose and sucrose as carbon sources for microorganisms.

Except for the 80 W treatment, the rest of treatments impacted the routes of flavone and flavanol biosynthesis and tyrosine metabolism. According to Widiez et al. (2011), they found the flavone 3,4-dihydroxybenzaldehyde, which is related to the biosynthesis of vanillin, coinciding with our study, as well as studies on the expression of genes associated with vanillin biosynthesis. In plants, through the shikimic acid pathway, tyrosine is produced, which is a precursor of ferulic acid, a molecule of major importance as it is part of the main vanillin precursors, including glucovanillin and vanillyl alcohol, being found in different stages and quantities during curing process (Cai et al, 2019; Gallage & Møller, 2015). With the above information, it has been found that these pathways directly and indirectly are related in the route of vanillin biosynthesis, presented by Walton et al. (2003), Sreedhar et al. (2007), Kaur and Chakraborty (2013) and Gu et al. (2017).

### 5.2 Targeted metabolomics

Based on the untargeted metabolomics, the targeted analysis was conducted for the identification and quantification of selected phenolics in *V. planifolia* pods in each killing treatment. For both metabolomic approaches, the control group is hot water immersion 80 °C treatment (80 W). It is desired to demonstrate if there is a relationship between the phenolic compounds profile of *V. planifolia* with killing treatments. Concerning the content of phenolic compounds found, a total of 15 phenolic compounds belonging to different classes were identified such as 8 phenolic acids, 2 flavonoids, 1 flavonol, 1 methoxybenzoic acid, 1 phenyl glycoside, 1 other polyphenol and the amino acid phenylalanine in *V. planifolia* pods samples (Table 1). The most abundant compounds found were vanillin with 1.89 % in FREE treatment and glucovanillin with 3.49 % in MIC treatment.

**Table 1:** Resulting phenolic compounds found in targeted metabolomics in four killing treatments: Hot water immersion (80 W), Microwave (MIC), Freezing (FREE) and Sonication (SON), showing the means and standard deviation of concentrations. Data are expressed in μg/g of dry tissue. R.t (Retention time). Rows with different letters mean that are statistically different by Tukey's test (*p* < 0.05).

Metabolite name	Class	Formula	R.t (min)	Treatments (μg/g)			
				FREE	MIC	SON	80 W
*L*-Phenylalanine	Amino acid	C_9_H_11_NO_2_	2.16	16.78 ± 0.84^a^	8.33 ± 0.25^b^	12.89 ± 0.48^c^	9.79 ± 0.13^d^
Protocatechuic acid	Phenolic acids	C_7_H_6_O_4_	2.91	18.07 ± 0.05^a^	8.76 ± 0.53^c^	9.51 ± 0.02^b^	8.36 ± 0.13^c^
4-Hydroxybenzoic acid	Phenolic acids	C_7_H_6_O_3_	4.38	237.96 ± 5.53^a^	77.07 ± 1.49^d^	113.05 ± 0.85^b^	98.05 ± 1.08^c^
Glucovanillin	Phenolic glycoside	C_14_H_18_O_8_	4.94	13,457.90 ± 758.84^b^	34,892.01 ± 630.68^a^	13,414.60 ± 233.32^b^	13,223.15 ± 330.50^b^
Vanillic acid	Phenolic acids	C_8_H_8_O_4_	5.93	369.24 ± 8.29^a^	214.52 ± 4.44^c^	293.60 ± 14.47^b^	239.53 ± 12.14^c^
Caffeic acid	Phenolic acids	C_9_H_8_O_4_	6.15	3.82 ± 0.36^a^	2.81 ± 0.25^b^	2.69 ± 0.29^b^	2.74 ± 0.08^b^
Procyanidin B2	Flavonols	C_30_H_26_O_12_	7.24	5.64 ± 0.18^a^	3.11 ± 0.06^c^	5.36 ± 0.22^a^	3.64 ± 0.25^b^
Vanillin	Other Polyphenols	C_8_H_8_O_3_	7.65	18,907.68 ± 727.53^a^	11,368.54 ± 435.17^d^	15,645.50 ± 1015.13^b^	13,338.94 ± 366.67^c^
4-Coumaric acid	Phenolic acids	C_9_H_8_O_3_	8.23	12.04 ± 0.15^a^	10.90 ± 0.23^b^	9.27 ± 0.10^d^	10.38 ± 0.22^c^
Ferulic acid	Phenolic acids	C_10_H_10_O_4_	9.69	46.16 ± 1.13^a^	6.95 ± 0.27^d^	32.07 ± 1.11^b^	15.43 ± 0.29^c^
Sinapic acid	Phenolic acids	C_11_H_12_O_5_	10.26	5.10 ± 0.64^a^	5.31 ± 0.23^a^	4.03 ± 0.30^b^	4.0 ± 0.22^b^
*p*-Anisic acid	Methoxybenzoic acid	C_8_H_8_O_3_	11.68	9.24 ± 0.21^a^	4.87 ± 0.42^c^	9.75 ± 0.34^a^	6.74 ± 0.49^b^
*trans*-Cinnamic acid	Phenolic acids	C_9_H_8_O_2_	15.31	2.90 ± 0.16^b^	1.19 ± 0.09^c^	4.03 ± 0.13^a^	1.45 ± 0.10^c^
Luteolin	Flavonoid	C_15_H_10_O_6_	16.33	3.45 ± 0.02^a^	2.65 ± 0.03^c^	2.06 ± 0.05^d^	2.78 ± 0.05^b^
Apigenin	Flavonoid	C_15_H_10_O_5_	18.68	15.53 ± 0.51^a^	10.44 ± 0.22^c^	15.34 ± 0.18^ab^	14.59 ± 0.21^b^

**Fig. 2:**
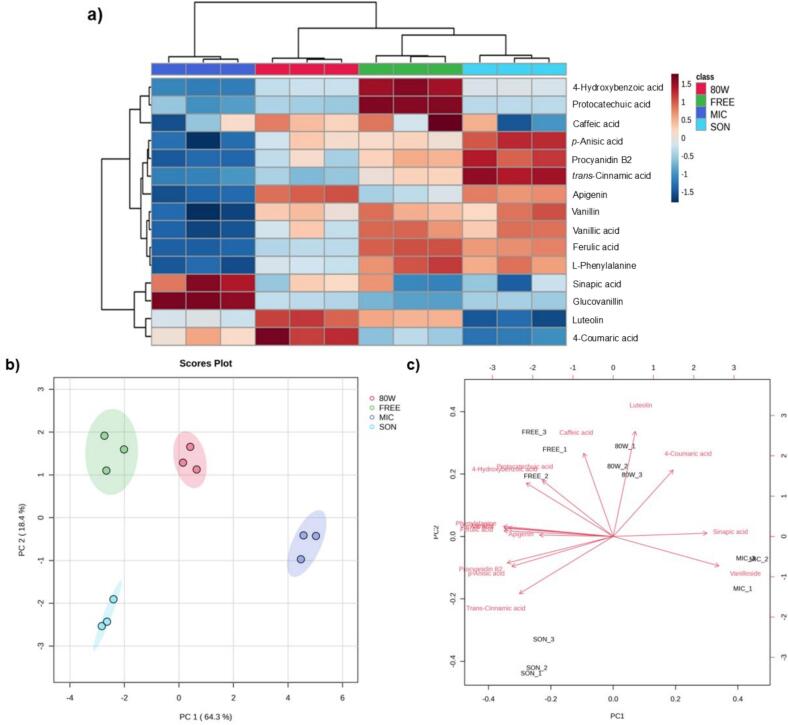
a) Heatmap representing the identified metabolites in targeted metabolomic analysis. High content of phenolic compounds are found in the FREE treatment. b) Principal Component Analysis (PCA) respecting to the metabolites obtained in the targeted analysis because of different killing treatments in *V. planifolia* pods. c) Biplot graph showing the main contributions of the 4 killing treatments in the found metabolites. Glucovanillin in MIC, luteolin in 80 W, protocatechuic acid and caffeic acid in FREE and *trans*-cinnamic acid in SON.

The heatmap (Fig. 2a), shows that the FREE treatment exhibited higher content of 4-hydroxybenzoic acid, protocatechuic acid and caffeic acid. The SON treatment obtained higher content of *p*-anisic acid, procyanidin B2 and *trans*-cinnamic acid. In the case of MIC treatment, exhibited higher content of sinapic acid and glucovanillin. Finally, the 80 W treatment presented higher contents of luteolin and 4-coumaric acid. This analysis was constructed based on the concentrations obtained, with a red *z-score* meaning that the average is 1.5 standard deviations above the average, meaning that, is the highest content and blue the lowest.

As can be seen in the Principal Component Analysis (PCA) (Fig. 2b), with an explained variance of 82.7 %, FREE and 80 W treatments clustered separately over the component 2. However, MIC and SON treatments clustered separately over the PCA 1, confirming for all cases that the content of phenolic compounds in each treatment are different. These targeted results confirmed the final grouping of phenolic compounds impacted by the killing treatments. In addition, the Biplot (Fig. 2c) shows that the metabolites with higher contributions in each treatment are glucovanillin in MIC, luteolin and 4-coumaric acid in 80 W, protocatechuic acid, 4-hydroxybenzoic acid and caffeic acid in FREE and *trans-*cinnamic acid in SON.

On the other hand, Antonio-Gutiérrez et al. (2023), carried out the killing and post-killing treatments similar to this research, varying their conditions. The pods were subjected to 80 W (3 min, 65 °C), MIC (2.5 min) and SON (3 min, 65 °C). Subsequently, the pods were treated with a post-killing process, by sonication at different times (5, 10, 15 min, 38 °C). Sweating, drying and conditioning stages were replaced by oven drying at 38 °C, reaching a weight loss of 70 %. The period of curing was carried out in 10, 20, 30 and 40 d. The vanillin concentrations obtained by Antonio-Gutiérrez et al. (2023), in 80 W, MIC and SON treatments, at 5 min post-killing condition, in 30 d were 1.56 %, 1.48 % and 2.68 % successively. In the research of Pardio et al. (2009), with 80 W and FREE treatments, they obtained 2.61 % and 2.84 % of vanillin respectively.

In this study, the killing treatment that obtained higher concentration of vanillin was FREE (1.89 %), followed by SON (1.56 %), 80 W (1.33 %) and MIC (1.14 %). This result agrees with what Pardio et al. (2009) mentioned, freezing treatment obtained higher content of vanillin, 4-hydroxybenzaldehyde, vanillic acid, vanillyl alcohol, causing mechanical damage to the subcellular structure and cell wall, leading to organelle rupture and decompartmentalization (Mariezcurrena et al., 2008).

The SON treatment obtained high percentage of vanillin, because sonication generates the greatest rupture of cell membranes, causing β-ᴅ-glucosidase and vanillin precursors to maintain high contact. It is important to mention that if the intensity of the sonication increased over 10 min, the yield of vanillin decreased, resulting in variations in enzyme activity (Antonio-Gutiérrez et al., 2023).

In the case of 80 W treatment that obtained lower concentration of vanillin, it has been found in traditional curing that after killing, the enzymatic activity is reduced by 50 %, meaning that half of the β-ᴅ-glucosidase reacted with glucovanillin, which is one of the main precursors of vanillin (Buitimea-Cantúa et al, 2022; Antonio-Gutiérrez et al, 2023). Other metabolites such as ferulic acid, vanillyl alcohol and *p*-coumaric acid are precursors that participate in the vanillin biosynthetic pathway, also some of these metabolites function as aroma precursors such as vanillin, vanillic acid and 4-hydroxybenzaldehyde (Cai et al., 2019).

For MIC treatment, it was the one that obtained the lowest concentration of vanillin, however, it had the highest concentration of its main precursor, glucovanillin (3.49 %). This can be explained by the loss of volatile compounds and aromatic compounds that can be lost by chemical degradation. Furthermore, Havkin-Frenkel & Belanger (2018) found out that severe killing conditions such as excessive or prolonged heat can cause complete destruction of beneficial enzymes, thus disrupting enzyme activity for biocatalysis of sensory compounds.

Besides Antonio-Gutiérrez et al. (2023), reported that on day 20 obtained high percentages of vanillin in MIC and SON treatments with a post-killing between 5 and 10 min. This can be explained by the fact that, in traditional curing, drying represents a physical effect and if drying time is prolonged, the vanillin content decreases. Another important point is that in the final stage of curing, some biochemical reactions are generated, such as oxidative degradation, where vanillin can be oxidized and lost (Zamora-Flores., 2015).

Regarding the method of extraction of phytochemicals, both Antonio-Gutiérrez et al. (2023) and Pardio et al. (2009), used ethanol as solvent, without mechanical manipulation. In this study, 100 % methanol was used, obtaining vanillin concentrations within the ranges reported in the literature. According to Jadhav et al. (2009), the solvent with the highest recovery of vanillin is ethanol, followed by methanol. However, it has been found in literature that the accelerated solvent extraction method is where the highest recovery of phytochemical compounds is obtained (96 %) (Zhang et al., 2022). In addition, this method consumes low amounts of solvents, implying time reductions, and simplifying extraction methods.

### 5.3 16S sequencing analysis

**Fig. 3:**
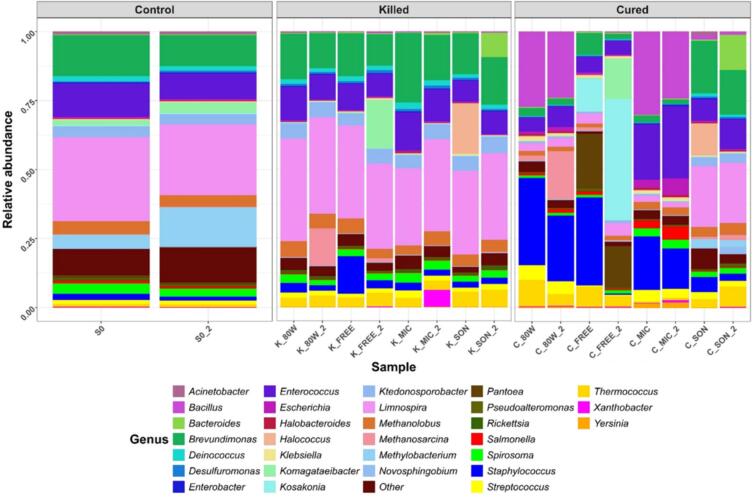
Relative abundances of bacterial populations in the 3 study groups of *V. planifolia* pods: Control (Ctrl), Killed pods (K), Cured pods (C). A high displacement of bacterial populations is obtained in Cured group.

In vanilla, different bacterial populations have been shown to contribute partial degradation of the cell wall, allowing enzyme-substrate contact for the formation of compounds responsible for the vanilla aroma (Barrientos et al., 2023). It can be seen in genus graph (Fig. 3), the control (Ctrl), *Limnospira* (28 %), *Enterococcus* (11 %) and *Brevundimonas* (13 %), are the genera with the highest relative abundance and a higher displacement, because the conditions of the green pods were not modified.

In contrast to killed group (K), a displacement was generally observed in *Komagataeibacter* and *Klebsiella* genus. For the KFREE treatment, the genus with the highest displacement was *Komagataeibacter* (18 %), while in KSON it was *Klebsiella* (0.50 %). As in the Ctrl group, the genera *Enterococcus, Brevundimonas* and *Limnospira* seem to be conserved despite the killing methods (KMIC, KSON, K80 W, KFREE). The different temperatures and conditions to which they were subjected caused cell rupture, assuming that this made bacterial genera resilient at these conditions, generating modification of other genera thanks to these changes.

However, a greater impact was found on the displacement of bacterial populations in cured group (C). *Limnospira* (22 %), *Brevundimonas* (18 %) and *Klebsiella* (0.43 %) were the genera that displaced the most. In CSON, *Bacillus* (26 %), *Staphylococcus* (23 %) are the majority in C80 W and CMIC, highlighting *Enterococcus* (23 %) in CMIC and *Methanosarcina* (10 %) in C80 W, *Kosakonia* (28 %), *Komagataeibacter* (15 %), *Staphylococcus* (16 %) and *Pantoea* (18 %) were found in CFREE. These considerably relevant displacement of bacterial populations was possibly to the later stages of vanilla curing, since following the killing, sweating, drying and conditioning are the next stages.

Thermal treatments in C group obtained significant changes in the α-diversity of vanilla pods (Table 2), which is associated to describe species richness within a functional community (Andermann et al., 2022). In observed ASVs, there was greater diversity in Ctrl and in C groups, compared to K group. Except for CSON, the C group presented greater Shannon equity, compared to Ctrl and K group. In contrast, the K group presented a greater Simpson dominance, compared against Ctrl and C. All these treatments were evaluated with *p* < 0.05, where in Chao1 no significant differences were obtained, that is, all the means were similar.

**Table 2:** Resulting α-diversities showing different means in Amplicon Sequence Variants (ASV), Chao 1, Shannon evenness and Simpson dominance indexes. Rows with different letters mean statistically different by Tukey's test (p < 0.05).

Treatments	ASV	Chao 1	Shannon evenness	Simpson dominance
**S0**	349 ± 5.66^a^	408.86 ± 5.86^a^	1.79 ± 0.05^bc^	0.37 ± 0.01^bc^
**K80 W**	264.5 ± 17.7^ab^	318.9 ± 21.5^a^	1.53 ± 0.07^cd^	0.41 ± 0.03^ab^
**KMIC**	212.5 ± 13.44^b^	246 ± 22.7^a^	1.42 ± 0.10^d^	0.44 ± 0.03^a^
**KSON**	227 ± 31.1^b^	263.6 ± 46.1^a^	1.60 ± 0.02^cd^	0.39 ± 0.01^abc^
**KFREE**	231.5 ± 10.61^b^	295.8 ± 17.8^a^	1.55 ± 0.07^cd^	0.40 ± 0.02^abc^
**C80 W**	302 ± 32.5^ab^	417.1 ± 32.6^a^	2.47 ± 0.11^a^	0.13 ± 0.01^e^
**CMIC**	255 ± 12.73^ab^	354.8 ± 35.9^a^	2.43 ± 0.04^a^	0.13 ± 0.001^e^
**CSON**	297.5 ± 37.5^ab^	353 ± 23.5^a^	1.87 ± 0.06^b^	0.34 ± 0.002^c^
**CFREE**	268 ± 52.3^ab^	382.6 ± 111.1^a^	2.40 ± 0.05^a^	0.19 ± 0.02^d^

**Fig. 4:**
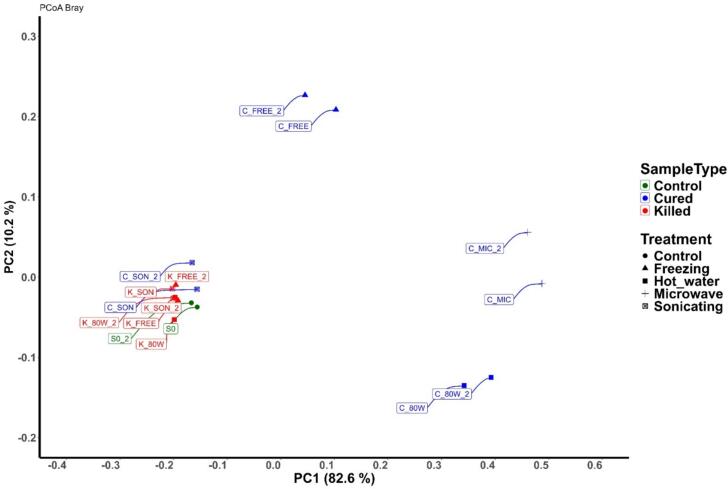
β-diversity PCoA Bray Curtis analysis. Thermal treatments like Microwave (CMIC), Hot water immersion 80 °C (C80 W) and Freezing (CFREE) are separated and dispersed in this plot, meaning differences in bacterial populations. CSON was clustered with the killed (K) treatments and the control, assuming similarity in their microorganisms.

For β-diversity measured by PCoA Bray Curtis (Fig. 4) which is associated to the differentiation between species communities (Andermann et al., 2022), the C treatments, especially CMIC, C80 W and CFREE, were grouped in different clusters, observing an important dissimilarity respecting to Ctrl and with those K pods on the PC1 axis. Also, the 4 treatments in K group and CSON pods were grouped with a similarity regarding the Ctrl on the PC2 axis. This was verified through a similarity analysis (ANOSIM) where the model revealed a significant difference (R^2^ = 0.485, *p* = 0.003) in the compositions of the bacterial communities of killing process. With these analyses, it is proposed that killing treatments with complete curing do generate changes in bacterial populations, due to both the dispersion of the clusters and bacterial diversity.

As mentioned above, sweating refers to the fermentation process of the pods, allowing in conditions of high humidity and temperature the activity of the enzymes glucosidase and oxidase for the development of aromatic compounds, while the pods acquire a dark brown color and a soft and flexible consistency (Zamora-Flores 2015). It can be suggested that greater dysbiosis of microorganisms occurred at this stage, due to fermentation and enzyme activity.

That could explain the study by Xu et al. (2020), where the bacterial genus that predominates in the role of vanilla aroma formation during curing process is *Bacillus*, having a relative abundance of 72.02 ± 19.77 % in the killing stage, with the activation of *Bacillus* strains in sweating, in drying the relative abundance abruptly decreases to 3.29 ± 1.76 % and in conditioning the relative abundance increases to 87.51 ± 6.48 %. Likewise, comparing to the other stages of curing, the relative abundance of *Lactococcus* in cured pods was much higher than in the other stages of curing with 41.69 ± 2.04 %, while in *Streptococcus* and *Pseudomonas* they obtained relative abundances of 3.37 ± 0.08 and 0.69 ± 0.04 % respectively, indicating that these three bacterial genera also play an important role in vanilla curing.

Likewise, Röling et al. (2001), reported that they obtained changes in the bacterial communities of green pods after the killing method, since in the green pods they obtained the genera *Enterobacter, Bacillus, Staphylococcus*, however, *Bacillus* (*B. subtilis, B. lincheniformis* and *B. smithii*) was the only genus that developed at high temperatures (up to 65 °C) during killing, remaining for more than a week after killing, due to its thermophilic and thermotolerant nature.

Similarly, Guevara et al. (2016), reported that bacterial communities found in green pods after 80 W killing are inhibited, but some microorganisms of *Bacillus* genus can persist, due to their thermophilic nature and spore production. Likewise, the pods with 80 W treatment had the presence of enterobacteria in later stages of curing process (Guevara et al., 2016). According to Barrientos et al. (2023), the dominant microorganisms found in different curing methods are in the genus *Bacillus* (*B. smithii, B. licheniformis, B. subtilis, B. pumilis and B. firmus*), because they showed thermoresistance during the different curing stages. These mentioned data support the results obtained in this approach, the C80 W and CMIC treatments were those that obtained the bacterial genus *Bacillus*, agreeing with Xu et al. (2020), Guevara et al. (2016), Barrientos et al. (2023), and Röling et al. (2001), although they differ in the percentages of abundances. Relative abundances obtained in K80 W and KMIC obtained relative abundances of 0.42 % and 0.26 % and in C80 W and CMIC 25.66 % and 27.27 % respectively. Also, the genera *Enterococcus* and *Streptococcus* are found in CTRL, K and C groups.

Respecting to α-diversity, according to Xu et al. (2020), with killing treatment of hot air at 70 °C in 5 min, they obtained higher Chao1 index when pods were killed (477.66), Shannon index 3.97 in drying step and Simpson index 0.33 in conditioning step. For this study the Shannon, Simpson and Chao1 indexes were different, assuming that different conditions that the pods were subjected were responsible for this bacterial diversity respecting to Xu et al. (2020) study.

### 5.4 Correlations between metabolomics and 16S sequencing

**Fig. 5:**
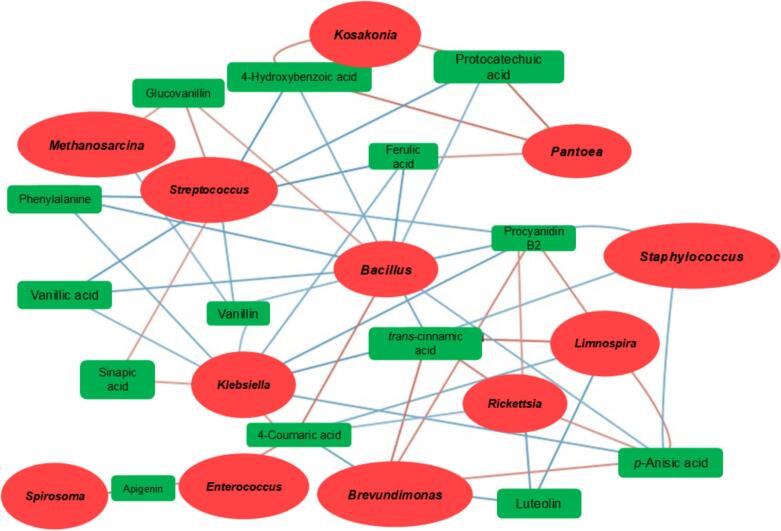
Interaction network of metabolomic and 16S Sequencing features for *V. planifolia*. Green squares represent metabolites found in targeted metabolomics; red circles represent bacteria; red and blue lines represent positive and negative correlations respectively. Those correlations were selected by *p* < 0.01. The interactions were visualized by Cytoscape software. (For interpretation of the references to color in this figure legend, the reader is referred to the web version of this article.)

In order to identify potential associations between phytochemical composition and bacterial populations, the relative abundances of bacteria were evaluated looking for correlations with the identified metabolites. The resulting network (Fig. 5) contained 26 nodes and 57 edges, represents the significant correlations between microorganisms and vanilla phenolic compounds. Significant positive correlations were found in *Bacillus* with glucovanillin and 4-coumaric acid (*p* < 0.01); *Klebsiella* with 4-coumaric acid and sinapic acid (p < 0.01); *Streptococcus* with glucovanillin and sinapic acid (p < 0.01) were those that obtained the highest correlations between 0.6 and 0.7. Significant negative correlations were found in *Brevundimonas* with 4-coumaric acid and luteolin (p < 0.01); *Bacillus* with vanillin, 4-hydroxybenzaldehyde, ferulic acid, vanillic acid and procyanidin B2 (p < 0.01) and *Streptococcus* with vanillin, vanillic acid, phenylalanine, ferulic acid and 4-hydroxybenzaldehyde (p < 0.01) were those that obtained highest correlations between −0.8 to −0.9.

Interestingly, glucovanillin was found to be highly positively correlated with genus *Bacillus*. This is consistent with reported by Chen et al. (2016) and Gu et al. (2017), where *Bacillus* is involved in the hydrolysis of glucovanillin and the direct formation of vanillin. They also suggested that β-ᴅ-glucosidase produced from *Bacillus* isolates, are involved in vanillin formation. Furthermore, Chen et al. (2016), reported that glucovanillin is dispersed from the inner to the outer part of vanilla pod during curing and is hydrolyzed by β-ᴅ-glucosidase. Also, enzymatic hydrolysis is generated on the surface of vanilla pods. It is suggested that glucovanillin correlates directly with *Bacillus*, i.e., the more Bacillus present, the more glucovanillin there is. However, vanillin has a negative correlation with *Bacillus*, because it is not a product that the microorganism consumes or metabolizes, a fact that can be corroborated in the network diagram. Positive correlations of *Kosakonia* and *Pantoea* with protocatechuic acid, 4-hydroxybenzoic acid and ferulic acid have also been found. Positive correlations of *Brevundimonas* with *trans*-cinnamic acid and *p*-anisic acid were also found. These correlations agree with those reported by Mahadeo et al. (2024), where they explored endophytic bacteria in green and cured pods in *V. planifolia*. The bacterial genera found in this research and reported by Mahadeo et al. (2024), in green and cured pods are *Pseudoalteromonas, Enterobacter, Acinetobacter, Klebsiella, Escherichia, Deinococcus, Bacteroides, Streptococcus, Pantoea, Kosakonia, Brevundimonas, Staphylococcus* and *Bacillus*.

Phenolic compounds released during the curing process serve as substrates for bacterial metabolism. Certain genera, such as *Bacillus* and *Pseudomonas*, possess enzymes like β-ᴅ-glucosidase, which enable the hydrolysis of glucovanillin into vanillin, directly influencing bacterial proliferation and dominance (Xu et al., 2020). Furthermore, the killing treatment induces the release of phenolic glucosides, creating a biochemical environment that affects bacterial colonization. According to Xu et al. (2020), bacterial richness and diversity decrease as the curing stages progress, coinciding with an increase in phenolic compound concentrations. These phenolics also shape the dominant bacterial profiles, highlighting their critical role in phenol metabolism and aroma formation. Xu et al. (2020) consider that phenolic compounds act as active modulators of bacterial community dynamics, serving as substrates for microbial metabolism while shaping the ecological conditions within vanilla pods.

According to Paul, Rai, Selvaraj, Srivastava, & Tripathi, 2021, the genus *Bacillus* is a potential producer of vanillin from ferulic acid, isoeugenol and eugenol, while Kaur and Chakraborty (2013) reported that there is a metabolic pathway for the conversion of eugenol to vanillin in *Bacillus* (*Bacillus cereus* strain PN24), and Chen et al. (2016), reported that *Bacillus subtilis* B7—S strain can form vanillin from the bioconversion of ferulic acid. It has been reported that *Klebsiella* produces the degradation of vanillin to vanillyl alcohol and vanillic acid, while *Staphylococcus* (*Staphylococcus aureus*) has the capacity to consume ferulic acid, accumulating 45.7 mg/L of vanillin on the second day (Kaur & Chakraborty 2013). As mentioned above, *Bacillus, Staphylococcus* and *Klebsiella* genera proved to influence vanillin formation. However, the other genera found in this study could contribute directly or indirectly in the formation of vanilla aromatic compounds. Therefore, their involvement should be further investigated in future work. For correlations between bacterial genera and tentative metabolites found in untargeted analysis, they can be seen in supplementary material Fig. S5.

In summary, through multi-omic analysis we found important data to explore the relationship between key bacterial species and their metabolites both in the killing and in the rest of the vanilla curing process. This approach offers new perspectives on the microorganisms that contribute to the production of metabolites with industrial and biological relevance. The background must also be laid for further study of microorganisms and their metabolic mechanisms of bioconversion to form vanilla phytochemical compounds at each stage of the curing process.

## 6 Conclusion

Finally, it can be said that this is the first time that a non-targeted and targeted metabolomics, as well as 16S sequencing approach has been performed to study the killing effect on vanilla pods, since four killing treatments and bacterial population of these treatments was not compared previously. Also, this study proved that different killing treatments affect the composition of phenolic compounds and bacterial populations in *V. planifolia*. Freezing treatment is being the killing method that obtained higher phytochemical profile, and higher vanillin concentration. β-diversity is more related to the changes of the temperature of the killing method. *Bacillus* genera had higher correlations with glucovanillin, causing it's hydrolyzation producing vanillin. In future studies, it will be recommended to perform the extraction of phytochemical compounds with ethanol, and to study the effect of replacing the stages of sweating, drying and conditioning with oven drying at 38 °C until 70 % of the weight is lost, since in previous lines higher concentrations of vanillin were obtained under these conditions. It is expected that with these multi-omic tools, vanilla curing can be technified and made easier for producers to carry out those methods, obtaining better quality vanilla extracts.

## Funding sources

This research was funded by CONAHCYT (Consejo Nacional de Humanidades, Ciencia y Tecnología) in the maintenance resource, Instituto Tecnológico de Monterrey for the Master's degree scholarship and The Institute for Obesity Research for the economic resources for reagents and analysis.

## CRediT authorship contribution statement

**Tiffany A. Cuan-Escobar:** Writing – original draft, Methodology, Investigation, Formal analysis, Data curation, Conceptualization. **Alma Cuellar-Sánchez:** Writing – review & editing, Supervision, Methodology, Data curation, Conceptualization. **Haiku D.J. Gómez-Velázquez:** Writing – review & editing, Methodology, Formal analysis, Data curation. **Juan L. Monribot-Villanueva:** Writing – review & editing, Methodology, Formal analysis, Data curation. **José A. Guerrero-Analco:** Writing – review & editing, Methodology, Formal analysis, Data curation. **Isabel Gutiérrez-Díaz:** Writing – review & editing, Data curation. **Diego A. Luna-Vital:** Writing – review & editing, Project administration, Investigation, Funding acquisition, Data curation, Conceptualization.

## Declaration of competing interest

The authors declare that they have no known competing financial interests or personal relationships that could have appeared to influence the work reported in this paper.

## Data Availability

Data will be made available on request.

## Appendix A Supplementary data

Supplementary material 1 Image 1
