# Supplementary material for: Effect of different killing methods during curing on the phytochemical and bacterial composition of Vanilla planifolia using multi-omic approaches
Source: Food Chem X. 2025 Feb 8;26:102269. doi: 10.1016/j.fochx.2025.102269 (PMC11872573; doi:10.1016/j.fochx.2025.102269)
Supplement: Supplementary file 1 — Supplementary material 1 [file mmc1.docx]

| **Compound** | **dMRM transition** | | | **Mass spectrometric conditions** | | | **Quantification conditions** | | |
| --- | --- | --- | --- | --- | --- | --- | --- | --- | --- |
|  | **Precursor ion** | **Product ion** | **Retention time** | **Collision energy** | **Fragmentor** | **Polarity** | **Quantification range (µM)** | **Regression type** | **R^2^** |
| Shikimic acid^1^ | 173.1 | 111.1 | 0.53 | 10 | 100 | Negative | 0.5 - 19 | Quadratic | 0.99 |
| Gallic acid^2^ | 169.0 | 125.2 | 1.56 | 10 | 100 | Negative | 0.5 - 19 | Quadratic | 0.99 |
| L-Phenylalanine^3^ | 166.1 | 131.0 | 2.16 | 10 | 100 | Positive | 0.5 - 19 | Quadratic | 0.99 |
| Protocatechuic acid^1^ | 153.0 | 109.1 | 2.91 | 10 | 100 | Negative | 0.5 - 19 | Quadratic | 0.99 |
| 4-Hydroxybenzoic acid^1^ | 137.1 | 92.8 | 4.38 | 10 | 100 | Negative | 0.5 - 19 | Quadratic | 0.99 |
| Gentisic acid^1^ | 153.0 | 109.0 | 4.44 | 10 | 100 | Negative | 0.5 - 19 | Quadratic | 0.99 |
| Vainilloside^2^ | 315.1 | 153.0 | 4.94 | 10 | 100 | Positive | 0.5 - 19 | Quadratic | 0.99 |
| 4-Hydroxyphenylacetic acid^3^ | 107.1 | 77.0 | 5.38 | 20 | 140 | Positive | 0.5 - 19 | Quadratic | 0.99 |
| (-)-Epigallocatechin^4^ | 305.1 | 125.0 | 5.44 | 20 | 140 | Negative | 0.5 - 19 | Quadratic | 0.99 |
| (+)-Catechin^2^ | 291.0 | 138.9 | 5.73 | 10 | 100 | Positive | 0.5 - 19 | Quadratic | 0.99 |
| Vanillic acid^1^ | 169.0 | 93.0 | 5.93 | 10 | 100 | Positive | 0.5 - 19 | Quadratic | 0.99 |
| Chlorogenic acid^1^ | 355.1 | 163.0 | 6.14 | 10 | 100 | Positive | 0.5 - 19 | Quadratic | 0.99 |
| Caffeic acid^1^ | 181.0 | 163.0 | 6.15 | 10 | 100 | Positive | 0.5 - 19 | Quadratic | 0.99 |
| Scopolin^5^ | 355.1 | 193.0 | 6.18 | 20 | 100 | Positive | 0.5 - 19 | Quadratic | 0.99 |
| Malvin^1^ | 655.1 | 331.1 | 6.42 | 40 | 100 | Positive | 0.5 - 19 | Quadratic | 0.99 |
| Kuromanin^1^ | 449.0 | 286.9 | 6.98 | 30 | 100 | Positive | 0.5 - 19 | Quadratic | 0.99 |
| Procyanidin B2^1^ | 577.1 | 425.1 | 7.24 | 10 | 100 | Negative | 0.5 - 19 | Quadratic | 0.99 |
| Keracyanin^2^ | 595.2 | 287.1 | 7.56 | 20 | 100 | Positive | 0.5 - 19 | Quadratic | 0.99 |
| Vanillin^1^ | 153.0 | 124.9 | 7.65 | 10 | 100 | Positive | 0.5 - 19 | Quadratic | 0.99 |
| (-)-Epicatechin^2^ | 291.0 | 138.8 | 7.68 | 10 | 100 | Positive | 0.5 - 19 | Quadratic | 0.99 |
| Mangiferin^2^ | 423.0 | 302.8 | 8.00 | 10 | 100 | Positive | 0.5 - 19 | Quadratic | 0.99 |
| 4-Coumaric acid^1^ | 165.0 | 147.0 | 8.23 | 10 | 100 | Positive | 0.5 - 19 | Quadratic | 0.99 |
| (-)-Gallocatechin gallate^2^ | 458.9 | 139.0 | 8.74 | 20 | 80 | Positive | 0.5 - 19 | Quadratic | 0.99 |
| Umbelliferone^1^ | 163.0 | 107.0 | 8.81 | 30 | 100 | Positive | 0.5 - 19 | Quadratic | 0.99 |
| Quercetin 3,4-di-O-glucoside^1^ | 627.0 | 302.9 | 9.51 | 10 | 100 | Positive | 0.5 - 19 | Quadratic | 0.99 |
| Scopoletin^1^ | 193.0 | 133.0 | 9.63 | 10 | 100 | Positive | 0.5 - 19 | Quadratic | 0.99 |
| Ferulic acid^1^ | 195.1 | 145.0 | 9.69 | 20 | 100 | Positive | 0.5 - 19 | Quadratic | 0.99 |
| 3-Coumaric acid^1^ | 165.05 | 147.04 | 9.78 | 10 | 100 | Positive | 0.5 - 19 | Quadratic | 0.99 |
| Sinapic acid^1^ | 225.1 | 207.1 | 10.26 | 10 | 100 | Positive | 0.5 - 19 | Quadratic | 0.99 |
| Salicylic acid^2^ | 137.0 | 93 | 10.70 | 10 | 100 | Negative | 0.5 - 19 | Quadratic | 0.99 |
| Epicatechin gallate^4^ | 443.1 | 123.0 | 10.71 | 10 | 100 | Positive | 0.5 - 19 | Quadratic | 0.99 |
| Ellagic acid^1^ | 300.5 | 145.0 | 10.72 | 30 | 170 | Negative | 0.5 - 19 | Quadratic | 0.99 |
| Myricitrin^1^ | 465.0 | 318.9 | 10.82 | 10 | 100 | Positive | 0.5 - 19 | Quadratic | 0.99 |
| Quercetin 3-D-galactoside^2^ | 465.0 | 302.9 | 11.09 | 10 | 100 | Positive | 0.5 - 19 | Quadratic | 0.99 |
| Rutin^2^ | 611.0 | 302.9 | 11.12 | 10 | 100 | Positive | 0.5 - 19 | Quadratic | 0.99 |
| Quercetin 3-glucoside^2^ | 465.0 | 303.0 | 11.38 | 10 | 100 | Positive | 0.5 - 19 | Quadratic | 0.99 |
| Luteolin 7-O-glucoside^1^ | 449.0 | 287.0 | 11.60 | 10 | 100 | Positive | 0.5 - 19 | Quadratic | 0.99 |
| *p*-Anisic acid^3^ | 153.1 | 109.0 | 11.68 | 5 | 120 | Positive | 0.5 - 19 | Quadratic | 0.99 |
| Penta-O-galloyl-B-D-glucose^2^ | 771.1 | 153.0 | 12.45 | 20 | 100 | Positive | 0.5 - 19 | Quadratic | 0.99 |
| 2,4-Dimethoxy-6-methylbenzoic acid^2^ | 197.0 | 179.0 | 12.57 | 5 | 80 | Positive | 0.5 - 19 | Quadratic | 0.99 |
| Kaemperol 3-O-glucoside^1^ | 449.0 | 286.9 | 12.76 | 10 | 100 | Positive | 0.5 - 19 | Quadratic | 0.99 |
| Quercitrin^1^ | 449.1 | 303.1 | 12.79 | 10 | 100 | Positive | 0.5 - 19 | Quadratic | 0.99 |
| Naringin^1^ | 273.0 | 153.0 | 12.91 | 10 | 120 | Positive | 0.5 - 19 | Quadratic | 0.99 |
| Myricetin^1^ | 317.0 | 179.0 | 13.16 | 10 | 100 | Negative | 0.5 - 19 | Quadratic | 0.99 |
| Hesperidin^1^ | 609.1 | 301.1 | 13.48 | 20 | 100 | Negative | 0.5 - 19 | Quadratic | 0.99 |
| Rosmarinic acid^1^ | 361.1 | 163.0 | 13.62 | 10 | 100 | Positive | 0.5 - 19 | Quadratic | 0.99 |
| *trans*-Resveratrol^2^ | 229.1 | 135.0 | 13.81 | 10 | 100 | Positive | 0.5 - 19 | Quadratic | 0.99 |
| Phloridzin^1^ | 435.0 | 272.9 | 13.84 | 10 | 100 | Negative | 0.5 - 19 | Quadratic | 0.99 |
| Secoisolariciresinol^2^ | 363.2 | 137.1 | 13.98 | 20 | 100 | Positive | 0.5 - 19 | Quadratic | 0.99 |
| *trans*-Cinnamic acid^1^ | 149.1 | 131.0 | 15.31 | 10 | 100 | Positive | 0.5 - 19 | Quadratic | 0.99 |
| Quercetin^2^ | 301 | 151 | 16.17 | 20 | 100 | Negative | 0.5 - 19 | Quadratic | 0.99 |
| Luteolin^1^ | 285 | 151 | 16.33 | 20 | 100 | Negative | 0.5 - 19 | Quadratic | 0.99 |
| Psoralen^1^ | 187.0 | 131.1 | 16.82 | 20 | 100 | Positive | 0.5 - 19 | Quadratic | 0.99 |
| Angelicin^2^ | 187.0 | 131.1 | 17.49 | 20 | 100 | Positive | 0.5 - 19 | Quadratic | 0.99 |
| Naringenin^1^ | 271.0 | 151 | 17.97 | 10 | 100 | Negative | 0.5 - 19 | Quadratic | 0.99 |
| Apigenin^1^ | 271.0 | 153.0 | 18.68 | 30 | 100 | Positive | 0.5 - 19 | Quadratic | 0.99 |
| Kaempferol^1^ | 285 | 151 | 18.99 | 10 | 100 | Negative | 0.5 - 19 | Quadratic | 0.99 |
| Hesperetin^1^ | 303.1 | 177.1 | 19.26 | 20 | 100 | Positive | 0.5 - 19 | Quadratic | 0.99 |
| Podophyllotoxin^4^ | 415.1 | 397.1 | 20.18 | 10 | 100 | Positive | 0.5 - 19 | Quadratic | 0.99 |
| Methyl cinnamate^2^ | 163.1 | 131.0 | 23.21 | 6 | 100 | Positive | 0.5 - 19 | Quadratic | 0.99 |
| Nordihydroguaiaretic acid^1^ | 303.0 | 193.1 | 23.73 | 10 | 100 | Positive | 0.5 - 19 | Quadratic | 0.99 |
| Chrysin^1^ | 255.1 | 153.0 | 24.37 | 40 | 100 | Positive | 0.5 - 19 | Quadratic | 0.99 |
| Kaempferide^1^ | 299 | 284 | 25.75 | 20 | 100 | Positive | 0.5 - 19 | Quadratic | 0.99 |
| Emodin^1^ | 269.0 | 225.0 | 28.79 | 20 | 150 | Negative | 0.5 - 19 | Quadratic | 0.99 |
| Chrysophanol^1^ | 255.1 | 153.0 | 32.47 | 40 | 100 | Positive | 0.5 - 19 | Quadratic | 0.99 |

**Table S1.** Chromatographic and spectrometric conditions for phenolics-targeted metabolomics. The retention time variation allowed for the search of the compounds were 2 min in each case. The cell accelerator voltage was 7 V for each compound. Dilutions were made if the concentration of some compounds were higher than the linearity range. Compounds were purchased from ^1^Extrasynthese (Lyon, France), ^2^Sigma Aldrich (St. Louis, USA), ^3^kindly donated by Dr. Thor Arnason (University of Ottawa), ^4^Cayman Chemical Company (Michigan, USA), ^5^isolated in-house.

| **Treatments** | **Retention time (min)** | **Mass/charge (Da)** | **Matched compounds** | **KEGG ID** | **Adducts** | **Mass difference (mDa)** |
| --- | --- | --- | --- | --- | --- | --- |
| T1- Microwave | 0.48 | 223.0255 | Sideretin | C22007 | [M-H]^-^ | 0.67646677 |
|  | 0.52 | 215.0356 | Methoxsalen | C01864 | [M-H]^-^ | 0.57646677 |
|  | 0.55 | 59.0139 | Acetic acid | C00033 | [M-H]^-^ | 0.07646677 |
|  | 0.55 | 59.0139 | Glycolaldehyde | C00266 | [M-H]^-^ | 0.07646677 |
|  | 0.56 | 321.0611 | trans-3,3',4',5,5',7-Hexahydroxyflavanone | C02906 | [M+H]^+^ | 0.62353323 |
|  | 1.3 | 134.0471 | Adenine | C00147 | [M-H]^-^ | 0.12353323 |
|  | 1.64 | 137.0243 | 4-Hydroxybenzoic acid | C00156 | [M-H]^-^ | 0.12353323 |
|  | 1.9 | 339.1068 | 3-O-p-Coumaroylquinic acid | C12208 | [M+H]^+^ | 0.67646677 |
|  | 1.91 | 341.1091 | Sucrose | C00089 | [M-H]^-^ | 0.17646677 |
|  | 1.91 | 341.1091 | Cellobiose | C00185 | [M-H]^-^ | 0.17646677 |
|  | 1.91 | 341.1091 | D-Maltose | C00208 | [M-H]^-^ | 0.17646677 |
|  | 1.91 | 341.1091 | Alpha-Lactose | C00243 | [M-H]^-^ | 0.17646677 |
|  | 1.91 | 341.1091 | Trehalose | C01083 | [M-H]^-^ | 0.17646677 |
|  | 1.91 | 341.1091 | beta-Cortol | C01235 | [M-H]^-^ | 0.17646677 |
|  | 1.91 | 341.1091 | Epimelibiose | C05400 | [M-H]^-^ | 0.17646677 |
|  | 1.91 | 341.1091 | Melibiose | C05402 | [M-H]^-^ | 0.17646677 |
|  | 1.98 | 193.0506 | Ferulic acid | C01494 | [M-H]^-^ | 0.02353323 |
|  | 1.98 | 193.0506 | 5-Hydroxyconiferaldehyde | C12204 | [M-H]^-^ | 0.02353323 |
|  | 2 | 167.0347 | Homogentisic acid | C00544 | [M-H]^-^ | 0.32353323 |
|  | 2 | 167.0347 | 3,4-Dihydroxymandelaldehyde | C05577 | [M-H]^-^ | 0.32353323 |
|  | 2.12 | 137.0239 | 4-Hydroxybenzoic acid | C00156 | [M-H]^-^ | 0.52353323 |
|  | 2.13 | 157.0504 | Isopropylmaleic acid | C02631 | [M-H]^-^ | 0.22353323 |
|  | 2.15 | 159.0293 | Oxoadipic acid | C00322 | [M-H]^-^ | 0.62353323 |
|  | 2.22 | 380.1562 | cis-Zeatin-7-N-glucoside | C16443 | [M-H]^-^ | 1.32353323 |
|  | 2.42 | 209.0452 | 5-Hydroxyferulic acid | C05619 | [M-H]^-^ | 0.32353323 |
|  | 2.42 | 165.0555 | Caffeyl alcohol | C12206 | [M-H]^-^ | 0.22353323 |
|  | 2.64 | 153.05483 | 3,4-Dihydroxyphenylacetaldehyde | C04043 | [M+H]^+^ | 0.25353323 |
|  | 2.64 | 153.05483 | ortho-Hydroxyphenylacetic acid | C05852 | [M+H]^+^ | 0.25353323 |
|  | 3.37 | 663.1106 | NAD | C00003 | [M-H]^-^ | 0.97646677 |
|  | 3.54 | 425.1778 | Chitobiose | C01674 | [M+H]^+^ | 1.22353323 |
|  | 3.58 | 337.0918 | 3-O-p-Coumaroylquinic acid | C12208 | [M-H]^-^ | 1.12353323 |
|  | 3.82 | 663.1119 | NAD | C00003 | [M-H]^-^ | 2.27646677 |
|  | 3.85 | 153.0548 | 3,4-Dihydroxyphenylacetaldehyde | C04043 | [M+H]^+^ | 0.22353323 |
|  | 3.85 | 153.0548 | ortho-Hydroxyphenylacetic acid | C05852 | [M+H]^+^ | 0.22353323 |
|  | 3.86 | 243.028 | Fucose 1-phosphate | C02985 | [M-H]^-^ | 0.47646677 |
|  | 3.87 | 285.0395 | Luteolin | C01514 | [M-H]^-^ | 0.92353323 |
|  | 3.87 | 285.0395 | Kaempferol | C05903 | [M-H]^-^ | 0.92353323 |
|  | 3.87 | 301.07057 | Homoeriodictyol chalcone | C16405 | [M-H]^-^ | 1.15353323 |
|  | 4.63 | 157.04999 | Isopropylmaleic acid | C02631 | [M-H]^-^ | 0.63353323 |
|  | 4.75 | 593.1496 | Kaempferol 3-O-rhamnoside-7-O-glucoside | C21854 | [M-H]^-^ | 1.62353323 |
|  | 5 | 129.0551 | Ketoleucine | C00233 | [M-H]^-^ | 0.62353323 |
|  | 5 | 455.1546 | 5,10-Methenyltetrahydrofolic acid | C00445 | [M-H]^-^ | 1.32353323 |
|  | 5 | 129.0551 | 3-Methyl-2-oxovaleric acid | C00671 | [M-H]^-^ | 0.62353323 |
|  | 5.31 | 593.1493 | Kaempferol 3-O-rhamnoside-7-O-glucoside | C21854 | [M-H]^-^ | 1.92353323 |
|  | 5.52 | 287.0548 | Aromadendrin | C00974 | [M-H]^-^ | 1.32353323 |
|  | 5.52 | 287.0548 | Fustin | C01378 | [M-H]^-^ | 1.32353323 |
|  | 5.52 | 287.0548 | Eriodictyol | C05631 | [M-H]^-^ | 1.32353323 |
|  | 5.52 | 287.0548 | Eriodictyol chalcone | C15525 | [M-H]^-^ | 1.32353323 |
|  | 5.66 | 157.0499 | Isopropylmaleic acid | C02631 | [M-H]^-^ | 0.72353323 |
|  | 6.36 | 337.0921 | 3-O-p-Coumaroylquinic acid | C12208 | [M-H]^-^ | 0.82353323 |
|  | 6.53 | 285.0394 | Luteolin | C01514 | [M-H]^-^ | 1.02353323 |
|  | 6.53 | 285.0394 | Kaempferol | C05903 | [M-H]^-^ | 1.02353323 |
|  | 6.53 | 301.0705 | Homoeriodictyol chalcone | C16405 | [M-H]^-^ | 1.22353323 |
|  | 6.95 | 285.0392 | Luteolin | C01514 | [M-H]^-^ | 1.22353323 |
|  | 6.95 | 285.0392 | Kaempferol | C05903 | [M-H]^-^ | 1.22353323 |
|  | 7.22 | 269.0444 | Apigenin | C01477 | [M-H]^-^ | 1.12353323 |
|  | 7.22 | 269.0444 | Norizalpinin | C10044 | [M-H]^-^ | 1.12353323 |
|  | 12.1 | 119.0498 | Phenylacetaldehyde | C00601 | [M-H]^-^ | 0.42353323 |
|  | 12.1 | 277.1226 | Pantetheine | C00831 | [M-H]^-^ | 0.12353323 |
|  | 13.95 | 347.1496 | Gibberellin A1 | C00859 | [M-H]^-^ | 0.42353323 |
|  | 13.95 | 347.1496 | Gibberellin A29 | C06096 | [M-H]^-^ | 0.42353323 |
|  | 13.95 | 347.1496 | Gibberellin A34 | C11868 | [M-H]^-^ | 0.42353323 |
|  | 14.18 | 255.2319 | Palmitic acid | C00249 | [M-H]^-^ | 1.02353323 |
|  | 14.18 | 449.2879 | 16-feruloyloxypalmitate | C18217 | [M+H]^+^ | 1.87646677 |

| **Treatment** | **Retention time (min)** | **Mass/charge (Da)** | **Matched compounds** | **KEGG ID** | **Adducts** | **Mass difference** |
| --- | --- | --- | --- | --- | --- | --- |
| T2- Hot water immersion | 0.53 | 377.0856 | Sucrose | C00089 | [M+Cl]^-^ | 5.00E-01 |
|  | 0.53 | 377.0856 | Cellobiose | C00185 | [M+Cl]^-^ | 5.00E-01 |
|  | 0.53 | 377.0856 | D-Maltose | C00208 | [M+Cl]^-^ | 5.00E-01 |
|  | 0.53 | 377.0856 | Alpha-Lactose | C00243 | [M+Cl]^-^ | 5.00E-01 |
|  | 0.53 | 281.0988 | Glycerophosphocholine | C00670 | [M+Na]^+^ | 1.08E+00 |
|  | 0.53 | 377.0856 | Trehalose | C01083 | [M+Cl]^-^ | 5.00E-01 |
|  | 0.53 | 377.0856 | beta-Cortol | C01235 | [M+Cl]^-^ | 5.00E-01 |
|  | 0.53 | 377.0856 | Epimelibiose | C05400 | [M+Cl]^-^ | 5.00E-01 |
|  | 0.53 | 377.0856 | Melibiose | C05402 | [M+Cl]^-^ | 5.00E-01 |
|  | 0.55 | 59.0139 | Acetic acid | C00033 | [M-H]^-^ | 7.65E-02 |
|  | 0.55 | 341.1084 | Sucrose | C00089 | [M-H]^-^ | 5.24E-01 |
|  | 0.55 | 341.1084 | Cellobiose | C00185 | [M-H]^-^ | 5.24E-01 |
|  | 0.55 | 341.1084 | D-Maltose | C00208 | [M-H]^-^ | 5.24E-01 |
|  | 0.55 | 341.1084 | Alpha-Lactose | C00243 | [M-H]^-^ | 5.24E-01 |
|  | 0.55 | 59.0139 | Glycolaldehyde | C00266 | [M-H]^-^ | 7.65E-02 |
|  | 0.55 | 341.1084 | Trehalose | C01083 | [M-H]^-^ | 5.24E-01 |
|  | 0.55 | 341.1084 | beta-Cortol | C01235 | [M-H]^-^ | 5.24E-01 |
|  | 0.55 | 341.1084 | Epimelibiose | C05400 | [M-H]^-^ | 5.24E-01 |
|  | 0.55 | 341.1084 | Melibiose | C05402 | [M-H]^-^ | 5.24E-01 |
|  | 0.58 | 503.1629 | Raffinose | C00492 | [M-H]^-^ | 1.18E+00 |
|  | 0.58 | 503.1629 | Dextrin | C00721 | [M-H]^-^ | 1.14E+00 |
|  | 0.58 | 503.1629 | D-Gal alpha 1->6D-Gal alpha 1->6D-Glucose | C05404 | [M-H]^-^ | 1.18E+00 |
|  | 2.63 | 247.0136 | Isopentenyl pyrophosphate | C00129 | [M+H]^+^ | 5.24E-01 |
|  | 2.63 | 247.0136 | Dimethylallylpyrophosphate | C00235 | [M+H]^+^ | 5.24E-01 |
|  | 13.95 | 347.1496 | Gibberellin A1 | C00859 | [M-H]^-^ | 4.24E-01 |
|  | 13.95 | 347.1496 | Gibberellin A29 | C06096 | [M-H]^-^ | 4.24E-01 |
|  | 13.95 | 347.1496 | Gibberellin A34 | C11868 | [M-H]^-^ | 4.24E-01 |
|  | 14.18 | 449.2879 | 16-feruloyloxypalmitate | C18217 | [M+H]^+^ | 1.88E+00 |

| **Treatment** | **Retention time (min)** | **Mass/charge (Da)** | **Matched compounds** | **KEGG ID** | **Adducts** | **Mass difference (mDa)** |
| --- | --- | --- | --- | --- | --- | --- |
| T3- Freezing | 0.48 | 223.0255 | Sideretin | C22007 | [M-H]^-^ | 0.67646677 |
|  | 0.52 | 215.0356 | Methoxsalen | C01864 | [M-H]^-^ | 0.57646677 |
|  | 0.55 | 59.0139 | Acetic acid | C00033 | [M-H]^-^ | 0.07646677 |
|  | 0.55 | 59.0139 | Glycolaldehyde | C00266 | [M-H]^-^ | 0.07646677 |
|  | 1.64 | 137.0243 | 4-Hydroxybenzoic acid | C00156 | [M-H]^-^ | 0.12353323 |
|  | 1.98 | 193.0506 | Ferulic acid | C01494 | [M-H]^-^ | 0.02353323 |
|  | 1.98 | 193.0506 | 5-Hydroxyconiferaldehyde | C12204 | [M-H]^-^ | 0.02353323 |
|  | 2 | 167.0347 | Homogentisic acid | C00544 | [M-H]^-^ | 0.32353323 |
|  | 2 | 167.0347 | 3,4-Dihydroxymandelaldehyde | C05577 | [M-H]^-^ | 0.32353323 |
|  | 2.13 | 157.0504 | Isopropylmaleic acid | C02631 | [M-H]^-^ | 0.22353323 |
|  | 2.15 | 159.0293 | Oxoadipic acid | C00322 | [M-H]^-^ | 0.62353323 |
|  | 2.63 | 151.0395 | 3,4-Dihydroxyphenylacetaldehyde | C04043 | [M-H]^-^ | 0.52353323 |
|  | 2.63 | 151.0395 | ortho-Hydroxyphenylacetic acid | C05852 | [M-H]^-^ | 0.52353323 |
|  | 3.37 | 663.1106 | NAD | C00003 | [M-H]^-^ | 0.97646677 |
|  | 3.87 | 285.0395 | Luteolin | C01514 | [M-H]^-^ | 0.92353323 |
|  | 3.87 | 285.0395 | Kaempferol | C05903 | [M-H]^-^ | 0.92353323 |
|  | 3.87 | 301.07053 | Homoeriodictyol chalcone | C16405 | [M-H]^-^ | 1.19353323 |
|  | 4.75 | 593.1496 | Kaempferol 3-O-rhamnoside-7-O-glucoside | C21854 | [M-H]^-^ | 1.62353323 |
|  | 6.53 | 285.0394 | Luteolin | C01514 | [M-H]^-^ | 1.02353323 |
|  | 6.53 | 285.0394 | Kaempferol | C05903 | [M-H]^-^ | 1.02353323 |
|  | 6.53 | 301.0705 | Homoeriodictyol chalcone | C16405 | [M-H]^-^ | 1.22353323 |
|  | 6.95 | 285.0392 | Luteolin | C01514 | [M-H]^-^ | 1.22353323 |
|  | 6.95 | 285.0392 | Kaempferol | C05903 | [M-H]^-^ | 1.22353323 |
|  | 7.22 | 269.0444 | Apigenin | C01477 | [M-H]^-^ | 1.12353323 |
|  | 7.22 | 269.0444 | Norizalpinin | C10044 | [M-H]^-^ | 1.12353323 |
|  | 10.6 | 311.2215 | 13-L-Hydroperoxylinoleic acid | C04717 | [M-H]^-^ | 1.32353323 |
|  | 10.6 | 311.2215 | 9(S)-HPODE | C14827 | [M-H]^-^ | 1.32353323 |
|  | 10.6 | 311.2215 | 9-Hydroxy-12-oxo-15(Z)-octadecenoic acid | C21924 | [M-H]^-^ | 1.32353323 |
|  | 12.1 | 277.1226 | Pantetheine | C00831 | [M-H]^-^ | 0.12353323 |
|  | 13.95 | 347.1496 | Gibberellin A1 | C00859 | [M-H]^-^ | 0.42353323 |
|  | 13.95 | 347.1496 | Gibberellin A29 | C06096 | [M-H]^-^ | 0.42353323 |
|  | 13.95 | 347.1496 | Gibberellin A34 | C11868 | [M-H]^-^ | 0.42353323 |
|  | 14.18 | 449.2879 | 16-feruloyloxypalmitate | C18217 | [M+H]^+^ | 1.87646677 |

| **Treatment** | **Retention time (min)** | **Mass/charge (Da)** | **Matched compound** | **KEGG ID** | **Adducts** | **Mass difference (mDa)** |
| --- | --- | --- | --- | --- | --- | --- |
| T4- Sonication | 0.48 | 223.0255 | Sideretin | C22007 | [M-H]^-^ | 0.67646677 |
|  | 0.52 | 215.0356 | Methoxsalen | C01864 | [M-H]^-^ | 0.57646677 |
|  | 0.53 | 281.0988 | Glycerophosphocholine | C00670 | [M-Na]^+^ | 1.07646677 |
|  | 0.55 | 59.0139 | Acetic acid | C00033 | [M-H]^-^ | 0.07646677 |
|  | 0.55 | 59.0139 | Glycolaldehyde | C00266 | [M-H]^-^ | 0.07646677 |
|  | 1.64 | 137.0243 | 4-Hydroxybenzoic acid | C00156 | [M-H]^-^ | 0.12353323 |
|  | 2 | 167.0347 | Homogentisic acid | C00544 | [M-H]^-^ | 0.32353323 |
|  | 2 | 167.0347 | 3,4-Dihydroxymandelaldehyde | C05577 | [M-H]^-^ | 0.32353323 |
|  | 2.15 | 159.0293 | Oxoadipic acid | C00322 | [M-H]^-^ | 0.62353323 |
|  | 2.63 | 151.0396 | 3,4-Dihydroxyphenylacetaldehyde | C04043 | [M-H]^-^ | 0.42353323 |
|  | 2.63 | 151.0396 | ortho-Hydroxyphenylacetic acid | C05852 | [M-H]^-^ | 0.42353323 |
|  | 3.37 | 663.1106 | NAD | C00003 | [M-H]^-^ | 0.97646677 |
|  | 3.87 | 285.0395 | Luteolin | C01514 | [M-H]^-^ | 0.92353323 |
|  | 3.87 | 285.0395 | Kaempferol | C05903 | [M-H]^-^ | 0.92353323 |
|  | 3.87 | 301.07054 | Homoeriodictyol chalcone | C16405 | [M-H]^-^ | 1.18353323 |
|  | 6.53 | 285.0394 | Luteolin | C01514 | [M-H]^-^ | 1.02353323 |
|  | 6.53 | 285.0394 | Kaempferol | C05903 | [M-H]^-^ | 1.02353323 |
|  | 6.53 | 301.0705 | Homoeriodictyol chalcone | C16405 | [M-H]^-^ | 1.22353323 |
|  | 7.22 | 269.0444 | Apigenin | C01477 | [M-H]^-^ | 1.12353323 |
|  | 7.22 | 269.0444 | Norizalpinin | C10044 | [M-H]^-^ | 1.12353323 |
|  | 13.95 | 347.1496 | Gibberellin A1 | C00859 | [M-H]^-^ | 0.42353323 |
|  | 13.95 | 347.1496 | Gibberellin A29 | C06096 | [M-H]^-^ | 0.42353323 |
|  | 13.95 | 347.1496 | Gibberellin A34 | C11868 | [M-H]^-^ | 0.42353323 |
|  | 14.18 | 449.2879 | 16-feruloyloxypalmitate | C18217 | [M+H]^+^ | 1.87646677 |

**Table S2.** Names of tentative compounds found in the four killing treatments (Freezing, Hot water immersion, Microwave, Sonication). The content of the table: Retention time (min), *m/z* (Da), KEGG ID, Adducts and Mass difference (mDa).

| **Treatment** | **Pathways** | **p.value** | **FDR** | **Impact** |
| --- | --- | --- | --- | --- |
| T1-Microwave | Flavonoid biosynthesis | 1.29E-07 | 1.17E-05 | 0.14047 |
|  | Flavone and flavonol biosynthesis | 0.00019828 | 0.0090218 | 0.35 |
|  | Galactose metabolism | 0.0015579 | 0.047257 | 0.33025 |
|  | Starch and sucrose metabolism | 0.0051321 | 0.11676 | 0.19846 |
|  | Phenylpropanoid biosynthesis | 0.012409 | 0.22584 | 0.1757 |
|  | Tyrosine metabolism | 0.017049 | 0.25858 | 0.1676 |
|  | Valine, leucine and isoleucine biosynthesis | 0.034352 | 0.44658 | 0.06197 |
| T2- Hot water immersion | Galactose metabolism | 1.77E-08 | 1.61E-06 | 0.41082 |
|  | Starch and sucrose metabolism | 5.63E-06 | 0.00025612 | 0.19846 |
| T3- Freezing | Flavone and flavonol biosynthesis | 1.89E-05 | 0.0017235 | 0.35 |
|  | Tyrosine metabolism | 0.0033276 | 0.075703 | 0.1676 |
| T4- Sonication | Flavone and flavonol biosynthesis | 0.00026684 | 0.015472 | 0.35 |
|  | Tyrosine metabolism | 0.0014192 | 0.04305 | 0.1676 |

**Table S3.** Resulting metabolic pathways of the four killing treatments (Freezing, Hot water immersion, Microwave and Sonication). For each treatment, the pathways are analyzed, including statistical values such as *p.value* (p<0.05), FDR (False Discovery Rate), which measures the correction for multiple testing and impact that reflects the relevance of the metabolic pathway in the context of the treatment.


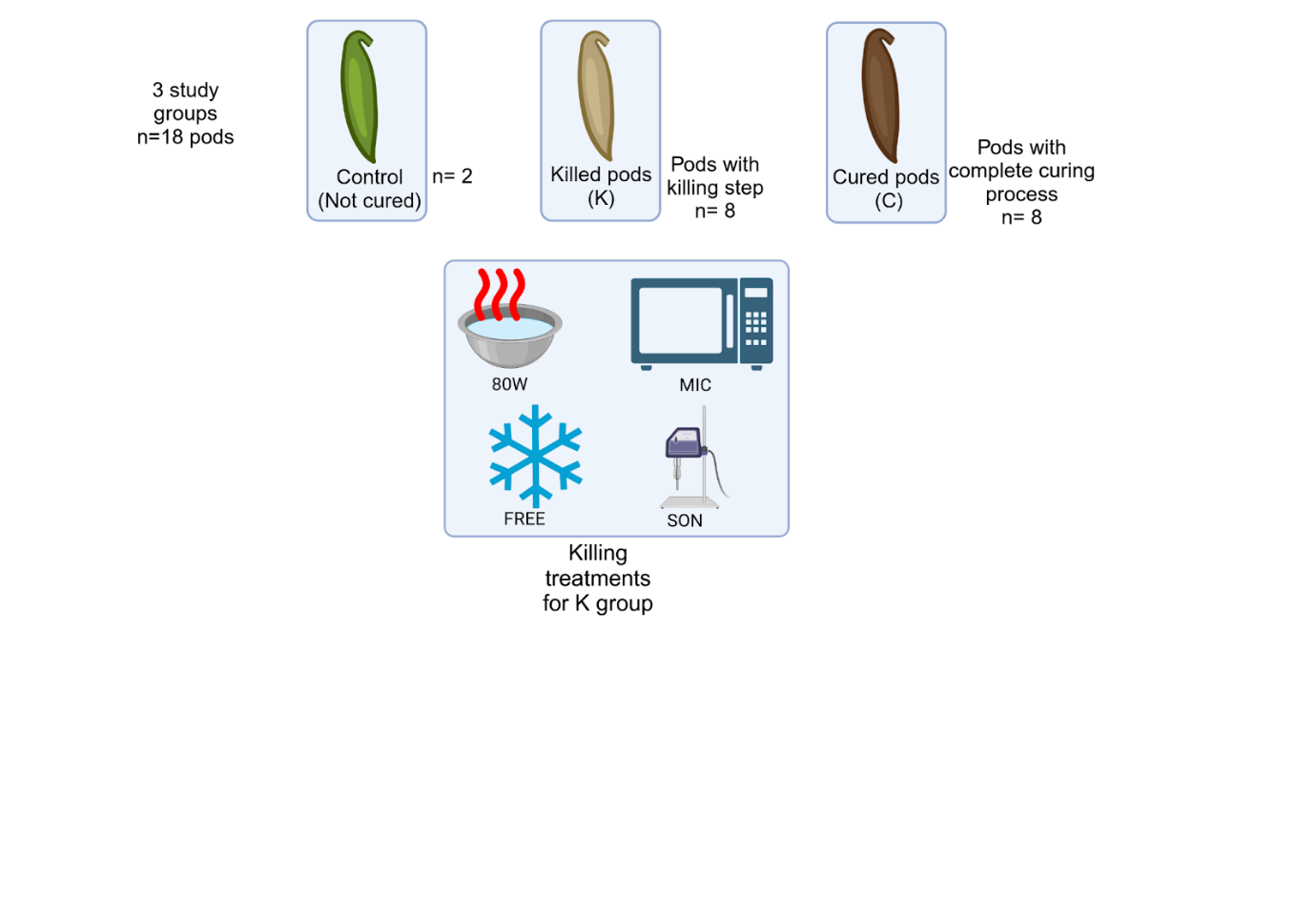


**Table S4.** Graphical design of experiment applied for the 16S sequencing experiment.

**
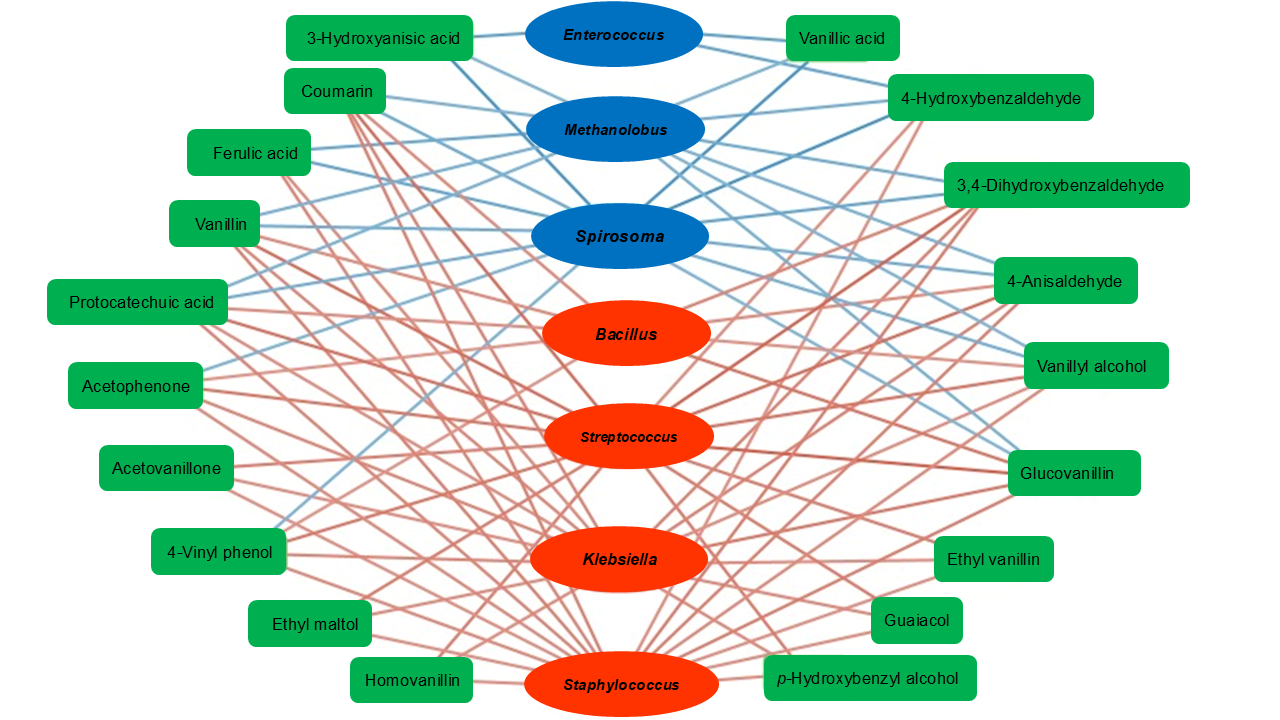
**

**Fig. S5.** Interaction network of untargeted metabolomic and 16S Sequencing features for *V. planifolia*. Green squares represent tentative metabolites; red circles represent bacteria with positive correlation; blue circles represent bacteria with negative correlations. Significant correlations were selected with a p<0.01. The interactions were generated and visualized using Cytoscape software.
